# Characterization of the SARS-CoV-2 co-receptor NRP1 expression profiles in healthy people and cancer patients: Implication for susceptibility to COVID-19 disease and potential therapeutic strategy

**DOI:** 10.3389/fgene.2022.995736

**Published:** 2022-10-19

**Authors:** Yongbiao Huang, Yuan Wang, Duo Xu, Lingyan Xiao, Wan Qin, Bo Liu, Xianglin Yuan

**Affiliations:** Department of Oncology, Tongji Hospital, Tongji Medical College, Huazhong University of Science and Technology, Wuhan, China

**Keywords:** NRP1, SARS-CoV-2, COVID-19, cancer, immune

## Abstract

Neuropilin-1 (NRP1) is a transmembrane protein involved in many physiological and pathological processes, and it functions as a co-receptor to facilitate the entry of SARS-CoV-2 into host cells. Therefore, it is critical to predict the susceptibility to SARS-CoV-2 and prognosis after infection among healthy people and cancer patients based on expression of NRP1. In the current study, we analyzed the conservation and isoform of NRP1 using public databases. NRP1 expression landscape in healthy people, COVID-19 patients, and cancer patients at both bulk and single-cell RNA-seq level was also depicted. We also analyzed the relationship between tissue-specific NRP1 expression and overall survival (OS), as well as tumor immune environment at a pan-cancer level, providing a comprehensive insight into the relationship between the vulnerability to SARS-CoV-2 infection and tumorigenesis. In conclusion, we identified NRP1 as a potential biomarker in predicting susceptibility to SARS-CoV-2 infection among healthy people and cancer patients.

## Introduction

Coronavirus diseases 2019 (COVID-19), caused by severe acute respiratory syndrome coronavirus 2 (SARS-CoV-2), broke out at the end of 2019 in Wuhan, China and quickly became pandemic worldwide (Holshue et al., 2020; Marchand-Senecal et al., 2020; Rothe et al., 2020; Zhu et al., 2020). According to Johns Hopkins University, a total of 528,430,289 confirmed cases and 6,286,507 deaths were reported globally as of 28 May 2022 (https://coronavirus.jhu.edu/).

Coronaviruses (CoVs), belonging to the family Coronaviridae, are enveloped viruses with a single-strand, positive-strand RNA (Su et al., 2016). Most CoVs only cause mild illness, but three could cause severe diseases in people, including severe acute respiratory syndrome coronavirus (SARS-CoV), Middle East respiratory syndrome coronavirus (MERS-CoV) and SARS-CoV-2 (Zheng, 2020). Symptoms caused by SARS-CoV-2 were more similar with that of SARS-CoV (Wang et al., 2020), and bioinformatics analysis revealed that the genome of SARS-CoV-2 has 82% nucleotide identity with SARS-CoV (Chan et al., 2020). The pathogenic mechanism of SARS-CoV-2 has not been fully elucidated. Researchers confirmed that SRAS-CoV-2 entered cells by the cell receptor angiotensin converting enzyme II (ACE2) (Zhou et al., 2020). Many proteins in host cells had been proven to interact with the spike glycoprotein on the outer membrane of SARS-CoV-2, thus contributed partially to the virulence of the virus, including furin (Walls et al., 2020), transmembrane protease serine 2 (TMPRSS2) (Hoffmann et al., 2020; Raghav et al., 2020), transmembrane protease serine 4 (TMPRSS4) (Zang et al., 2020), Glucose Regulated Protein 78 (GRP78) (Ibrahim et al., 2020), cathepsin L (CTSL) (Zhao et al., 2021), dipeptidyl peptidase 4 (DPP4) (Vankadari and Wilce, 2020), and neuropilin-1 (NRP1) (Daly et al., 2020).

NRP1, also known as CD304 or BDCA-4, is a member of the type I transmembrane protein family Neuropilins. NRP1 consists of an intracellular cytoplasmic domain, a transmembrane domain and an extracellular domain (Chaudhary et al., 2014), and the extracellular domain could be further divided into three parts, including CUB (a1/a2) domain, FV/FVIII (b1/62) domain, and MAM 3) domain (Gu et al., 2002). The MAM domain, together with the transmembrane domain, is necessary for the induction of neuropilin signaling (Roth et al., 2008). NRP1 acts as a co-receptor for some extracellular ligands to take part in a variety of physiological and pathological processes, including angiogenesis, cardiovascular development, immunity, cell migration, axonal guidance, and tumor development (Chaudhary et al., 2014). Recently, NRP1 was shown to serve as an entry factor and potentiate SARS-CoV-2 infectivity in multiple cell lines (Cantuti-Castelvetri et al., 2020; Daly et al., 2020). Generally speaking, the interaction between NRP1 and SARS-CoV-2 is based on the S1 CendR motif, which is generated by the furin cleavage of SARS-CoV-2 spike (S) protein (Daly et al., 2020). Blocking this interaction with a small-molecule inhibitor or monoclonal antibodies could reduce viral infection (Cantuti-Castelvetri et al., 2020; Daly et al., 2020; Kolarič et al., 2022), which provides a potential therapeutic target for COVID-19.

Cancer patients were found to have a higher COVID-19 morbidity and severity rate than individuals without cancer (Liang et al., 2020), partially owing to impaired immune systems (Liu C. et al., 2020). Main molecules intersecting COVID-19 and cancer included tumor necrosis factor α (TNF-α) (Balkwill, 2009; Huang et al., 2020), exportin 1 (XPO1) (Uddin et al., 2020; Azmi et al., 2021), Bruton tyrosine kinase (BTK) (Byrd et al., 2016; Roschewski et al., 2020), TMPRSS2 (Lin et al., 1999; Hoffmann et al., 2020; Raghav et al., 2020), NKG2A (André et al., 2018; Antonioli et al., 2020), and C5aR (Wang et al., 2019; Woodruff and Shukla, 2020). Common features shared by these molecules were all indispensable for immune responses against both cancer and infectious diseases. As NRP1 also plays an important role in immunity, cancer pathogenesis, and infection of SRAS-CoV-2, it is meaningful to explore the expression of NRP1 in different normal and cancer tissues to predict the susceptibility to COVID-19. Besides, the role NRP1 plays in the immunity microenvironment also needs exploration to better understand the pathogenesis of COVID-19.

Clinical management of COVID-19 patients mainly consisted of supportive therapy and symptoms treatment. Antiviral drugs including remdesivir, favipiravir, lopinavir-ritonavir, and camostat mesilate have already showed apparent efficacy in treating COVID-19 (Liu X. et al., 2020; Pascarella et al., 2020). Antibody drugs have been emerging as promising COVID-19 therapeutic agents and some of them have already been approved or received emergency use authorization (EUA) from the US Food and Drug Administration (FDA). SARS-CoV-2 neutralizing antibodies target the receptor binding domain (RBD) and N-terminal domain (NTD) on S1 protein, and S2 protein (Li et al., 2022). Besides, monoclonal antibodies to control the cytokine storm syndrome (CSS) are currently under evaluation in several clinical trials (Hwang et al., 2022). Taking these into account, we wondered whether antibody cocktails targeting NRP1 could benefit COVID-19 patients, especially patients complicated with cancer.

In this study, we demonstrated the landscape of NRP1 expression among different tissue types and did a pan-cancer analysis to figure out potential vulnerable people to COVID-19 using public databases. We further analyzed the relationship between NRP1 and immunity to interpret the immune disorders observed in severe cases of COVID-19. We hope our research could emphasize NRP1 as a reliable biomarker in predicting vulnerability to SARS-CoV-2 infection in cancer patients and provide potential strategy to treat cancer patients combined with COVID-19.

## Materials and methods

### Pathway analysis

The SARS-CoV-2 infection related pathways involving NRP1 were obtained from WikiPathways (https://www.wikipathways.org/index.php/Pathway:WP5065, https://www.wikipathways.org/index.php/Pathway:WP4846). WikiPathways is an open, collaborative platform dedicated to the curation of biological pathways (Martens et al., 2021).

### Homology analysis

Sequence alignment of NRP1 homologs were performed at the online website PRofile ALIgNEment (PRALINE) (https://www.ibi.vu.nl/programs/pralinewww/) (Simossis and Heringa, 2005), which integrates homology-extended and secondary structure information for multiple sequence alignment. The FASTA format file of NRP1 protein sequence in different species were downloaded from the NCBI database and it was uploaded to the website as the input file. All the parameters were set at default ones.

### HPA database analysis

The mRNA and protein expression levels of NRP1 in different human normal and tumor tissues were obtained from the Human Protein Atlas (HPA) database (https://www.proteinatlas.org/ENSG00000099250-NRP1) (Uhlén et al., 2015). The HPA consisted of 10 different sections, including tissue, brain, single cell type, tissue cell type, pathology, immune cell, blood protein, subcellular, cell line and metabolic. The mRNA expression data of NRP1 in normal and tumor tissues were derived from the consensus dataset (combined HPA and GTEx transcriptomics datasets), the FANTOM5 dataset, and the Cancer Genome Atlas (TCGA) dataset. The NRP1 protein expression data was from immunohistochemistry (IHC) analysis stained with two antibodies (cat #: HPA030278 and cat #: CAB004511). The NRP1 protein levels in plasma was detected by mass spectrometry and proximity extension assay (PEA). The single cell RNA sequencing data of lung tissues was derived from the Gene Expression Omnibus (GEO) dataset GSE130148. Data were accessed on 10 February 2022.

### Differential expression analysis in pan-cancers

The mRNA expression levels of NRP1 in different types of tumor tissues and paired normal tissues from the TCGA dataset were compared using the UALCAN (http://ualcan.path.uab.edu/index.html) and the ENCORI (https://starbase.sysu.edu.cn/panCancer.php) databases. The UALCAN database is a comprehensive and interactive web resource for analyzing gene expression data based on four databases including TCGA (Chandrashekar et al., 2017). The ENCORI database contained pan-cancer gene expression data of more than 30 types of tumors and provides gene differential analysis between tumor tissues and normal tissues (Shu et al., 2021). Gene mutation analysis of NRP1 across 25 cancer types was conducted using the Tumor Immune Estimation Resource (TIMER) 2.0 database (http://timer.comp-genomics.org/). TIMER2.0 provides tools to compare the differential gene expression between different mutation status (Li et al., 2020). Data were accessed on 10 February 2022.

### TISCH database analysis

The Tumor Immune Single Cell Hub (TISCH) is an online scRNA-seq database integrating single-cell transcriptomic data of nearly two million cells from 76 tumor datasets across 27 cancer types in 18 organs and tissues, focusing on tumor microenvironment (TME) (Sun et al., 2021). The NRP1 expression in non-small cell lung cancer (NSCLC) at the single-cell level were analyzed in the TISCH database (http://tisch.comp-genomics.org/home/). Data were accessed on 10 February 2022.

### GEPIA2 database analysis

GEPIA2 (Gene Expression Profiling Interactive Analysis 2) is a website providing tools including differential expression analysis, correlation analysis, profiling plotting, similar gene detection, survival analysis, and dimensionality reduction analysis (Tang et al., 2019). The NRP1 isoform usage, expression distribution and domain structures in multiple tumor tissues were determined by the GEPIA2 database (http://gepia2.cancer-pku.cn/#isoform) based on the large TCGA and Genotype-Tissue Expression (GTEx) datasets. The impact of NRP1 expression on overall survival (OS) of cancer patients were also determined *via* GEPIA2 (http://gepia2.cancer-pku.cn/#survival). Overall survival rate is defined as the percentage of people in a study or treatment group who are still alive for a certain period of time after they were diagnosed with or started treatment for cancer. Patients were classified into low NRP1 group and high NRP1 group based on the median NRP1 expression level for comparison of OS. Data were accessed on 10 February 2022.

### Correlation analysis of NRP1 expression and immune characteristics

The relations between NRP1 expression and the abundance of 28 immune cell types, immunoinhibitors, chemokines, and receptors across 30 cancer types were analyzed in the TISIDB database (http://cis.hku.hk/TISIDB/browse.php?gene=NRP1). The Tumor and Immune System Interaction Database (TISIDB) integrated multiple data resources in oncoimmunology to provide analysis on tumor-immune interactions (Ru et al., 2019). The distribution of NRP1 expression across different immune subtypes in pan-cancers was also analyzed in the TISIDB database. The cancer immune subtypes are divided into six categories, including C1 (wound healing), C2 (IFN-gamma dominant), C3 (inflammatory), C4 (lymphocyte depleted), C5 (immunologically quiet) and C6 (TGF-β dominant). Correlation between NRP1 and immune infiltrating cells in lung squamous cell carcinoma (LUSC) were further verified in the TIMER database (https://cistrome.shinyapps.io/timer/). Tumor Immune Estimation Resource (TIMER) integrated molecular profiles of tumor-immune interactions across 10,897 tumors from 32 cancer types (Li et al., 2017). Correlation between NRP1 and various immune checkpoint markers were also analyzed in the TIMER database. Data were accessed on 10 February 2022.

### Bulk RNA-seq and snRNA-seq analysis of NRP1 expression in COVID-19 patients

The GEO dataset GSE159585 contains bulk RNA-seq and single nucleus RNA-seq (snRNA-seq) data performed on lungs from seven COVID-19 patients and non-COVID-19 controls. Related data were downloaded from the GEO database (https://www.ncbi.nlm.nih.gov/geo/query/acc.cgi?acc=GSE159585). For bulk RNA-seq analysis, raw counts data was transformed into log2 (TPM+1) for comparison of gene expression. For snRNA-seq analysis, R package “Seurat” was used to process the raw counts data. Cells were classified into 21 clusters at a resolution of 0.2 after filtering out low low-quality cells.

### Analysis of NRP1 expression in human cancer cell lines

The normal human lung epithelial cell line BEAS-2b, the normal human colon cell line NCM460, the human lung adenocarcinoma cell line PC-9 and four colon cancer cell lines (HCT116, HT29, LoVo, and SW480) were obtained from the oncology laboratory of Tongji Hospital, Wuhan, China. BEAS-2b and PC-9 were cultured in RPMI-1640 medium (Hyclone, USA), HCT116 was cultured in McCoy’s 5A medium (Procell, China), and the rest cells were cultured in DMEM medium (HyClone, USA). Medium used contained 10% FBS (Gibco, USA) and all the cells were maintained in the incubator at 37°C supplemented with 5% CO_2_.

Total RNA from cells was isolated using TRIzol (Takara, Japan) and reverse-transcribed to cDNA with Hi Script II QRT SuperMix (Vazyme, China). Then, qRT-PCR was carried out in Real-Time PCR System (7900HT, Applied Biosystems, USA) using ChamQ universal SYBR qPCR Master Mix (Vazyme, China). The relative gene expression levels were calculated using the 2−ΔΔCT method, and the GAPDH mRNA expression levels were used for normalization. The primer sequences were listed as follows: NRP1-forward, 5′- CCC​CAA​ACC​ACT​GAT​AAC​TCG -3′ and NRP1- reverse, 5′- AGA​CAC​CAT​ACC​CAA​CAT​TCC -3'; GAPDH-forward, 5′- GAC​AGT​CAG​CCG​CAT​CTT​CT -3′ and GAPDH-reverse, 5′- GCG​CCC​AAT​ACG​ACC​AAA​TC -3'.

### Statistical analysis

Comparison of two normally distributed quantitative data were performed using the Student’s t test. Comparisons of multiple independent ordinal data were performed using Kruskal–Wallis test. Survival data were analyzed using Kaplan-Meier method and log-rank test. Spearman correlation test was applied to calculate the correlation between two normally distributed data. Two-side *p* < 0.05 was considered statistically significant. R software (version 4.2.0) and GraphPad Prism (version 8.0.1) were used for statistical analyses.

## Results

### NRP1 is involved in SARS-CoV-2 infection

To determine the role NRP1 plays in the infection process of SARS-CoV-2, we searched the biological pathways involving NRP1 in the WikiPathways database. Two pathways were determined, namely SARS-CoV-2 and COVID-19 pathway (WP4846) and SARS-CoV-2 altering angiogenesis *via* NRP1 (WP5065). Both of these two pathways indicated that NRP1 could act as a co-receptor when SARS-CoV-2 infects host human cells. Similar to ACE2, SARS-CoV-2 enters cells through the binding of surface spike glycoprotein (S protein) to NRP1 on the cell membrane (Supplementary Figure S1A). Additionally, SARS-CoV-2 could also facilitate angiogenesis *via* NRP1 (Supplementary Figure S1B). These results suggested that NRP1 plays an important role in SARS-CoV-2 infection and tumor growth.

### NRP1 is highly conserved

Alignment result of NRP1 homologs in different species including human (NP_003864.5), chimpanzee (XP_001143690.1), Rhesus monkey (NP_001252745.1), cow (NP_001192589.1), dog (XP_005617003.1), mouse (NP_032763.2), rat (NP_659566.1), chicken (NP_990113.1), frog (NP_001093692.1), and zebrafish (NP_852474.2) revealed that it was highly conserved (Supplementary Material S1), with a sequence identity percent reaching 83%. These indicated that NRP1, similar to ACE2 (Yan et al., 2020), would have the potential to act as a receptor for RBD on SARS-CoV-2 across different species of animals.

### Expression of NRP1 in healthy human tissues

**FIGURE 1:**
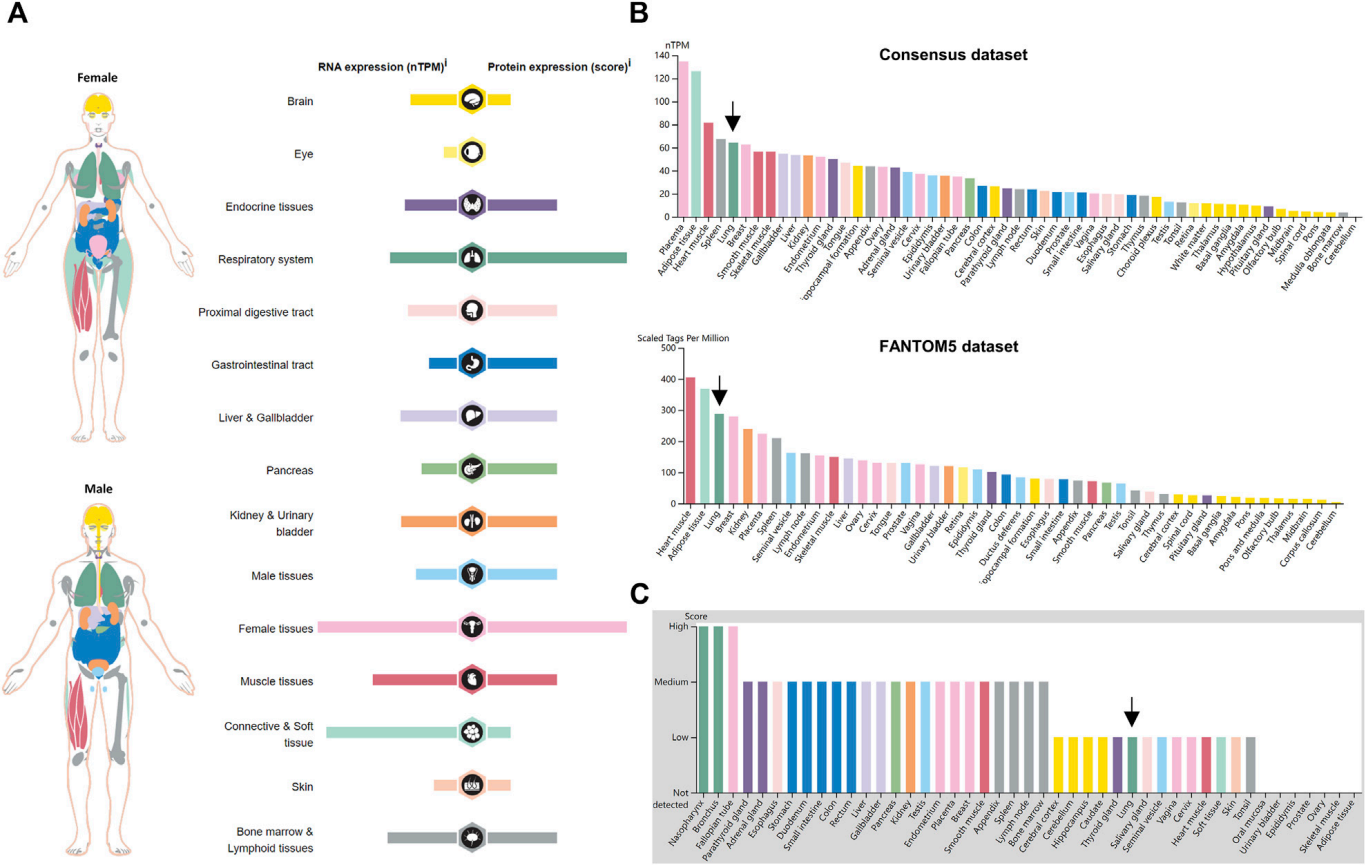
NRP1 expression in normal human tissues **(A)** The expression and distribution features of NRP1 in normal human tissues. **(B)** The mRNA expression profile of NRP1 in the Consensus dataset and FANTOM5 dataset ranked by expression levels. **(C)** The protein expression profile of NRP1 ranked by expression levels. Arrows indicated lung tissues.

An overview of NRP1 expression profiling at gene transcriptional and translational levels in different normal human tissues and organs was downloaded from the HPA database and was displayed in Figure 1A. We found that NRP1 showed low tissue specificity in mRNA expression levels. NRP1 was highly expressed in female tissues, connective and soft tissue, muscle tissues, respiratory system, kidney and urinary bladder, liver and gallbladder, and bone marrow and lymphoid tissues than other organs and tissues. The expression tendency of NRP1 protein was similar to that of mRNA, but respiratory system, gastrointestinal tract and pancreas showed higher translation level than transcription level. The transcription landscapes of NRP1 in multiple normal organs and tissues from the HPA and GTEx datasets were displayed respectively (Supplementary Figure S2). The mRNA expression data of NRP1 from the consensus dataset combining the HPA and GTEx dataset indicated that NRP1 expression in placenta is at the highest (134.8 nTPM), followed by adipose tissue (126.3 nTPM), heart muscle (81.5 nTPM), spleen (67.3 nTPM), lung (64.3 nTPM), and breast (62.7 nTPM) (Figure 1B). The cerebellum exhibited the lowest NRP1 expression (3.8 nTPM) among all the organs (Figure 1B). Similar mRNA expression distribution of NRP1 was observed in the FANTOM5 dataset (Figure 1B). As for the translation levels of NRP1, the IHC results revealed that nasopharynx, bronchus, and fallopian tube had highest protein expression among all the tissues. Twenty-one tissues had medium protein expression, fourteen tissues including the lung had low protein expression, and NRP1 protein expression was not detectable in seven tissues (Figure 1C). Together, these findings demonstrated that NRP1 is expressed at different transcription and translation levels in different human tissues and organs, with especially high expression in the respiratory system, indicating the susceptibility of the cells in the respiratory system to be infected by SARS-CoV-2.

### Expression of NRP1 in healthy human lungs

SARS-CoV-2 is mainly transmitted by droplet and aerosol, leading to severe acute respiratory syndrome (Jayaweera et al., 2020). Thus, the expression of SARS-CoV-2 receptors in the lung cells is crucial for viral entry into human body. The average mRNA expression level of lung tissues based on 578 samples from the GTEx dataset was 48.0 nTPM (Supplementary Figure S3A), and the max mRNA expression level of lung tissues based on the FANTOM5 project was 288.1 scaled tags per million (Supplementary Figure S3B). Notably, the mRNA level of NRP1 (64.3 nTPM) was nearly 80-fold higher than that of ACE2 (0.8 nTPM) in normal lung tissues from the consensus dataset (Supplementary Figure S3C). Single-cell analysis of lung tissues in HPA showed that NRP1 mRNA expression in macrophages is at the highest (94.1 nTPM), followed by endothelial cells (59.3 nTPM), alveolar cells type 2 (48.5 nTPM), and fibroblasts (47.0 nTPM) (Supplementary Figure S4A). Consistent with this result, IHC staining showed low to medium cytoplasmic and membranous NRP1 signals in macrophages, and none to low cytoplasmic and membranous NRP1 signals in alveolar cells (Supplementary Figure S4B). Comparison of the expression of NRP1 and ACE2 in healthy lung tissues revealed that NRP1 expression was significantly higher than that of ACE2 at both mRNA and protein level (Supplementary Figure S5). These findings indicated that, in addition to ACE2, NRP1 may also play an indispensable role in the invasion of lung tissues by SARS-CoV-2. Besides, macrophages and alveolar cells might be the main targets of SARS-CoV-2 in healthy people.

### Expression of NRP1 in lungs of COVID-19 patients

To compare NRP1 expression level between COVID-19 patients and non-COVID-19 people, the GEO dataset GSE159585 was analyzed. RNA-seq data revealed that the NRP1 expression level was significantly higher in lungs from COVID-19 patients than non-COVID-19 controls (*p* = 0.00067) (Supplementary Figure S6A). The same result was also obtained at the single-cell level (Supplementary Figure S6B). These indicated that people with high expression of NRP1 were vulnerable to SARS-CoV-2 infection and NRP1 could serve as a biomarker in predicting susceptibility to SARS-CoV-2 infection.

### NRP1 protein concentration in plasma

It was reported that COVID-19 patients were characterized with changes in peripheral white blood cells and immune cells (Chen et al., 2020), so we also analyzed the protein concentration of NRP1 in plasma and compared it with that of ACE2 *via* HPA. NRP1 protein concentration in plasma detected by mass spectrometry was 780 μg/L (Supplementary Figure S7A), nearly 2000-fold higher than that of ACE2 (400 ng/L) (Supplementary Figure S8). Besides, we found no difference in plasma NRP1 protein concentration between male and female detected by PEA (Supplementary Figure S7B). This result indicated that NRP1 might be involved in the pathogenesis of cytokine storm.

### Expression profile of NRP1 in malignant tumors

**FIGURE 2:**
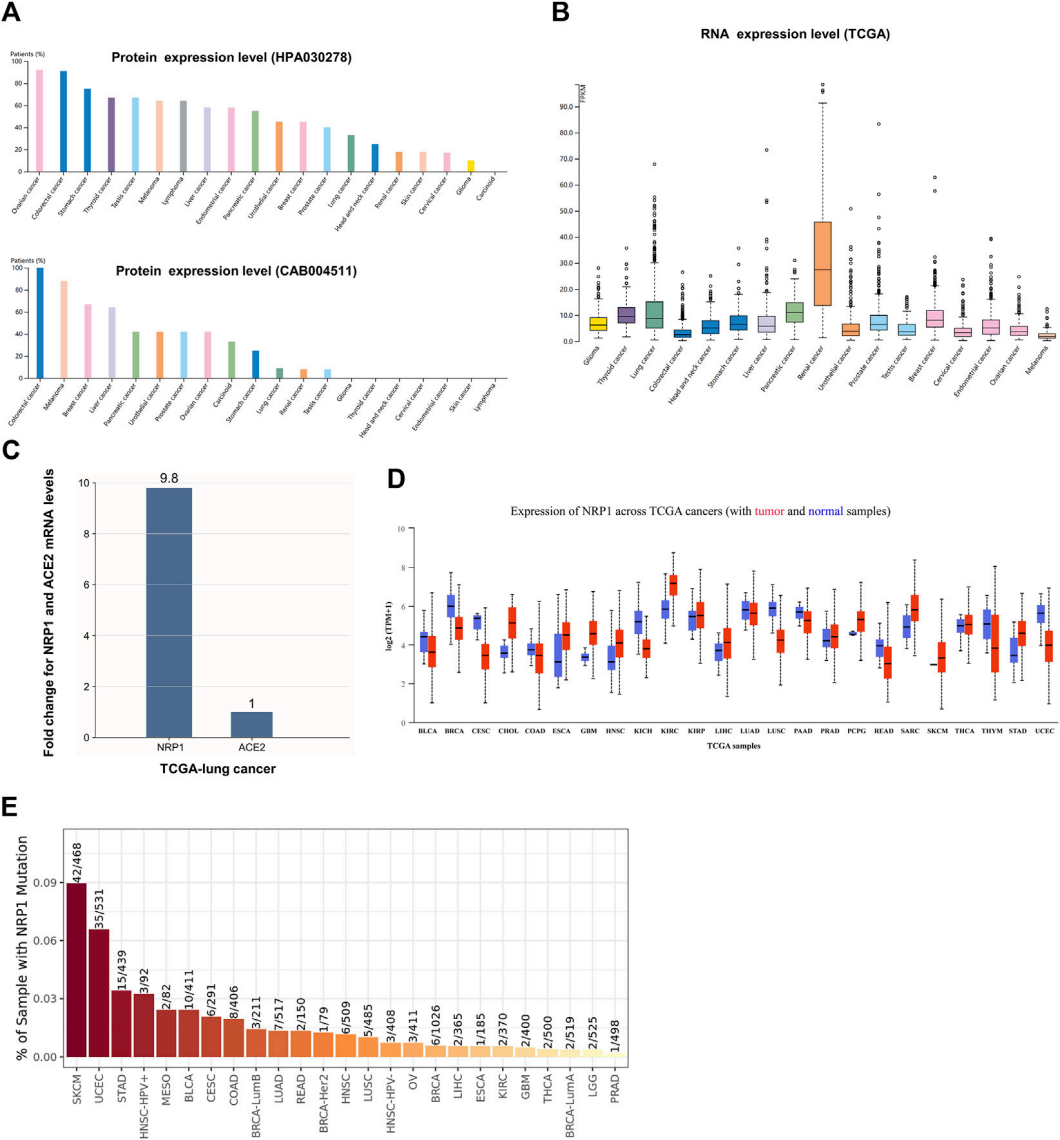
NRP1 expression in different cancer types **(A)** The protein expression profile of NRP1 ranked by expression levels. NRP1 proteins were detected by HPA030278 and CAB004511 respectively. **(B)** The pan-cancer RNA expression profile of NRP1 in the TCGA dataset **(C)** The mRNA expression level of NRP1 relative to that of ACE2 in the TCGA-lung cancer tissues. **(D)** The mRNA expression profile of NRP1 in tumor and normal samples across TCGA cancers. **(E)** Bar plot presenting NRP1 mutation frequency in the indicated TCGA cancer types.

Next, we estimated the expression profiles of NRP1 across pan-cancers. Analysis in the HPA database showed that the NRP1 transcription level was different from the translation level. NRP1 protein was highly expressed in ovarian cancer and colorectal cancer in the HPA030278 dataset, and was also highly expressed in colorectal cancer in the CAB004511 dataset (Figure 2A). NRP1 mRNA expression level showed low cancer specificity across pan-cancers, and was highest in renal cancer among all the cancer subtypes in the TCGA dataset (Figure 2B). Further analysis showed that the mRNA expression level of NRP1 (8.8 FPKM) was 9.8-fold higher than that of ACE2 (0.9 FPKM) in the TCGA-lung cancer dataset (Figure 2C). The mRNA expression landscape of NRP1 in normal and cancer samples across 24 TCGA cancer types was obtained from UALCAN and was shown in Figure 2D. Differential expression analysis of NRP1 at transcription level between normal tissues and cancer tissues across 17 different cancer types was carried out using ENCORI and the result was listed in Table 1. Generally speaking, the mRNA expression of NRP1 was significantly up-regulated in seven cancer types, including kidney renal clear cell carcinoma (KIRC), stomach adenocarcinoma (STAD), cholangiocarcinoma (CHOL), head and neck squamous cell carcinoma (HNSC), liver hepatocellular carcinoma (LIHC), thyroid carcinoma (THCA), and esophageal carcinoma (ESCA). Meanwhile, the mRNA expression of NRP1 was significantly down-regulated in five cancer types, including breast invasive carcinoma (BRCA), lung squamous cell carcinoma (LUSC), uterine corpus endometrial carcinoma (UCEC), kidney chromophobe (KICH), and colon adenocarcinoma (COAD). *In vitro* experiments validated this result. We found that relative NRP1 mRNA expression level was significantly lower in both the human lung adenocarcinoma cell line PC-9 and four human colon cancer cell lines compared with normal human cell lines (Supplementary Figure S9). NRP1 mutation analysis across different tumor types was carried out in TIMER2.0. The result showed that skin cutaneous melanoma (SKCM) had the highest mutation rate (8.97%) among all the cancer types, while prostate adenocarcinoma (PRAD) had the lowest mutation rate (0.20%) (Figure 2E). Above results indicated that mRNA expression level, protein expression level, and mutation rate of NRP1 in different cancer types varied a lot.

**TABLE 1:** The mRNA expression of NRP1 in different cancer types compared with normal tissues.

Cancer	Cancer full name	Cancer number	Normal number	Cancer Exp	Normal Exp	Fold change	*p* Value	FDR
BRCA	Breast Invasive Carcinoma	1104	113	9.21	19.58	0.47	1.3e-38	2.4e-37
LUSC	Lung Squamous Cell Carcinoma	501	49	6.47	17.88	0.36	1.9e-31	3.7e-30
KIRC	Kidney Renal Clear Cell Carcinoma	535	72	41.38	19.34	2.14	8.0e-23	8.2e-22
UCEC	Uterine Corpus Endometrial Carcinoma	548	35	6.40	14.73	0.43	8.0e-13	1.1e-11
STAD	Stomach Adenocarcinoma	375	32	7.45	4.11	1.81	5.6e-7	3.9e-6
KICH	Kidney Chromophobe	65	24	6.31	12.84	0.49	2.3e-6	1.2e-5
CHOL	Cholangiocarcinoma	36	9	14.39	3.99	3.61	0.00031	0.0012
HNSC	Head and Neck Squamous Cell Carcinoma	502	44	5.87	4.09	1.44	0.00031	0.0013
COAD	Colon Adenocarcinoma	471	41	3.68	4.46	0.83	0.00038	0.0015
LIHC	Liver Hepatocellular Carcinoma	374	50	7.73	4.48	1.73	0.001	0.0032
THCA	Thyroid Carcinoma	510	58	10.20	8.03	1.27	0.0021	0.0061
ESCA	Esophageal Carcinoma	162	11	6.85	4.28	1.60	0.0078	0.048
LUAD	Lung Adenocarcinoma	526	59	16.92	16.66	1.02	0.15	0.26
BLCA	Bladder Urothelial Carcinoma	411	19	5.83	5.87	0.99	0.15	0.36
KIRP	Kidney Renal Papillary Cell Carcinoma	289	32	16.66	15.29	1.09	0.18	0.71
PRAD	Prostate Adenocarcinoma	499	52	8.56	6.87	1.25	0.42	0.63
PAAD	Pancreatic Adenocarcinoma	178	4	11.67	15.61	0.75	0.45	0.66

Exp, Expression level; FDR, false discovery rate.

### NRP1 isoform usage and structure across pan-cancers

A novel ACE2 isoform had been reported to expresse in the airway epithelium and contribute to host susceptibility to SARS-CoV-2 (Blume et al., 2021). Thus, we wanted to figure out whether isoforms of NRP1, another SARS-CoV-2 receptor, had similar characteristics. GEPIA2 was used to analyze NRP1 (ENSG00000099250.17) isoform prevalence and structure in 33 tumor types. Fourteen isoforms of NRP1 were differentially expressed in tumor tissues (Supplementary Figure S10B), Usage of the isoform ENST00000374875.5 (NRP1-002) was the highest among all the isoforms, followed by the isoform ENST00000374867.6 (NRP1−202) (Supplementary Figure S10B). Usage of other isoforms was relatively low (Supplementary Figure S10B). Isoform structure prediction showed that encoding by 923 amino acids, NRP1-001 and NRP1-202 both had two CUB domains, two F5_F8_type_C domains, one MAM domain, and one DUF3481 domain (Supplementary Figure S10C). The rest isoforms all lacked certain segments (Supplementary Figure S10C). Taking this into account, we speculated that NRP1-202 might play an important role in tumorigenesis and SARS-CoV-2 infection.

### Comparison of NRP1 and ACE2 expression levels in tumor and matched normal tissues

Comparisons between two SARS-CoV-2 receptors, NRP1 and ACE2, in 23 different tumor and matched normal tissues were conducted using the TCGA dataset in GEPIA2. The result showed that the mRNA expression of NRP1 is higher than that of ACE2 in almost all the cancer types and matched normal tissues (Supplementary Figure S11), indicating that NRP1 might facilitate SARS-CoV-2 infection in both healthy people and cancer patients.

### Expression of NRP1 in lungs of NSCLC patients

**FIGURE 3:**
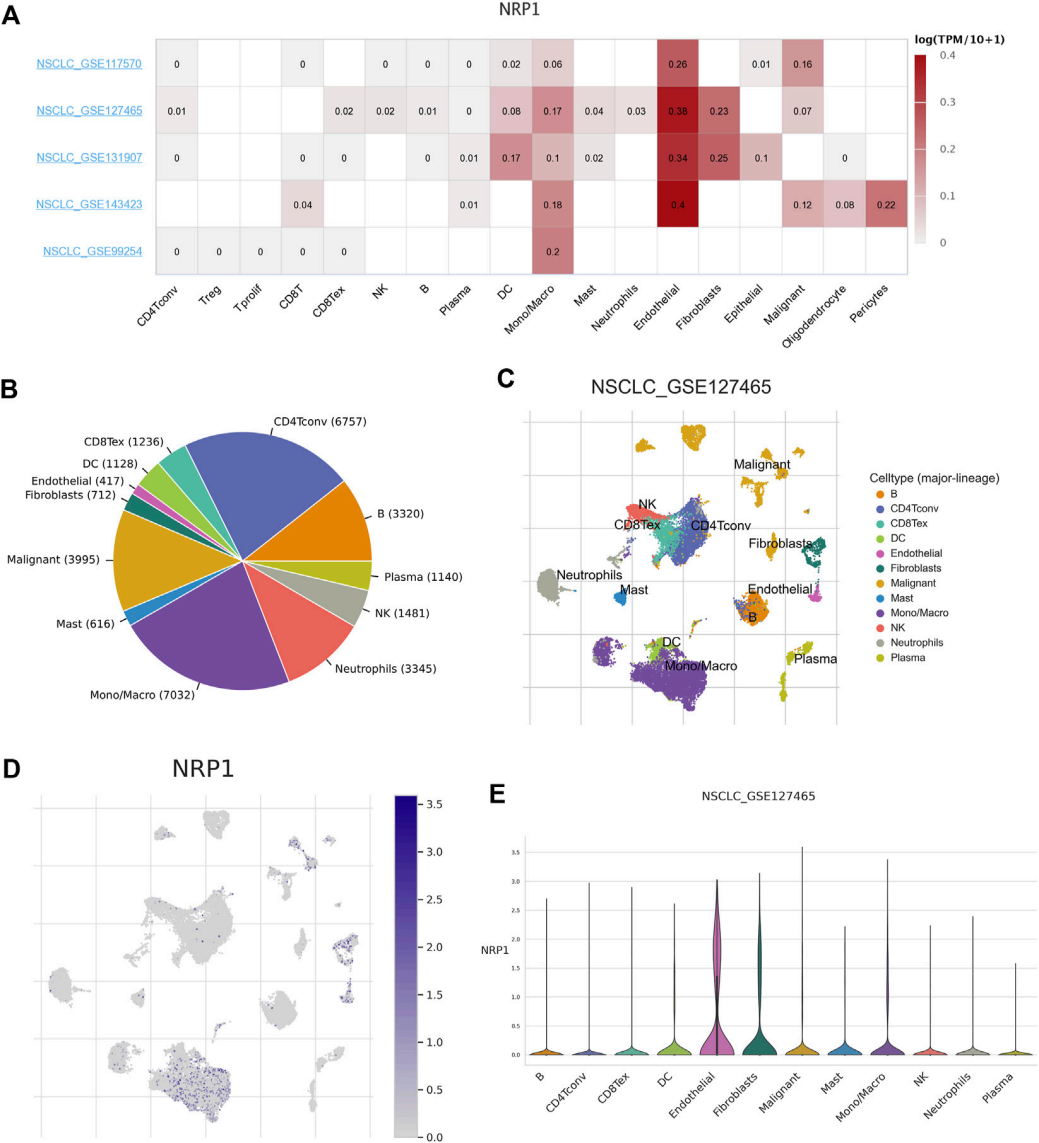
Correlation between NRP1 expression and TME in NSCLC **(A)** The heatmap showing the correlation between NRP1 expression level and TME in NSCLC tissues. **(B)** Pie chart showing the number of each cell type in the NSCLC_GSE127465 dataset **(C)** The distribution of each cell type in the NSCLC_GSE127465 dataset. **(D)** Heatmap showing the expression distribution of NRP1 across different cell types in the NSCLC_GSE127465 dataset. **(E)** The violin plot showing NRP1 expression of different cell types in the NSCLC_GSE127465 dataset.

As COVID-19 is mainly characterized by severe symptoms in the respiratory system, we wanted to find out the expression profile of the SARS-CoV-2 receptor NRP1 among NSCLC patients. Five datasets (i.e., NSCLC_GSE117570, NSCLC_GSE127465, NSCLC_GSE131907, NSCLC_GSE143423, and NSCLC_GSE99254) in the TISCH database were used to analyze the expression of NRP1 in various different cell types. Analysis combining the five datasets showed that NRP1 mRNA expression level was the highest in endothelial cells (Figure 3A). We further analyzed the NSCLC_GSE127465 dataset. This dataset contained 31,179 cells in total, and monocytes/macrophages (*n* = 7032) were the most abundant cells among them (Figure 3B). Cells in the NSCLC_GSE127465 dataset could be divided into 12 types based on marker genes (Supplementary Figure S12), including conventional CD4^+^ T cells, B cells, plasma cells, natural killer (NK) cells, neutrophils, monocytes/macrophages, mast cells, malignant cells, fibroblasts, endothelial cells, dendritic cells (DCs), and exhausted CD8^+^ T cells (Figure 3C). NRP1 mRNA expression distribution across all the cells was shown in Figure 3D. NRP1 mRNA expression was the highest in endothelial cells among all the cell subtypes, followed by fibroblasts and monocytes/macrophages (Figure 3E). This result indicated that endothelial cells might be the main target of SARS-CoV-2 among NSCLC patients.

### Prognostic significance of NRP1 in pan-cancers

**FIGURE 4:**
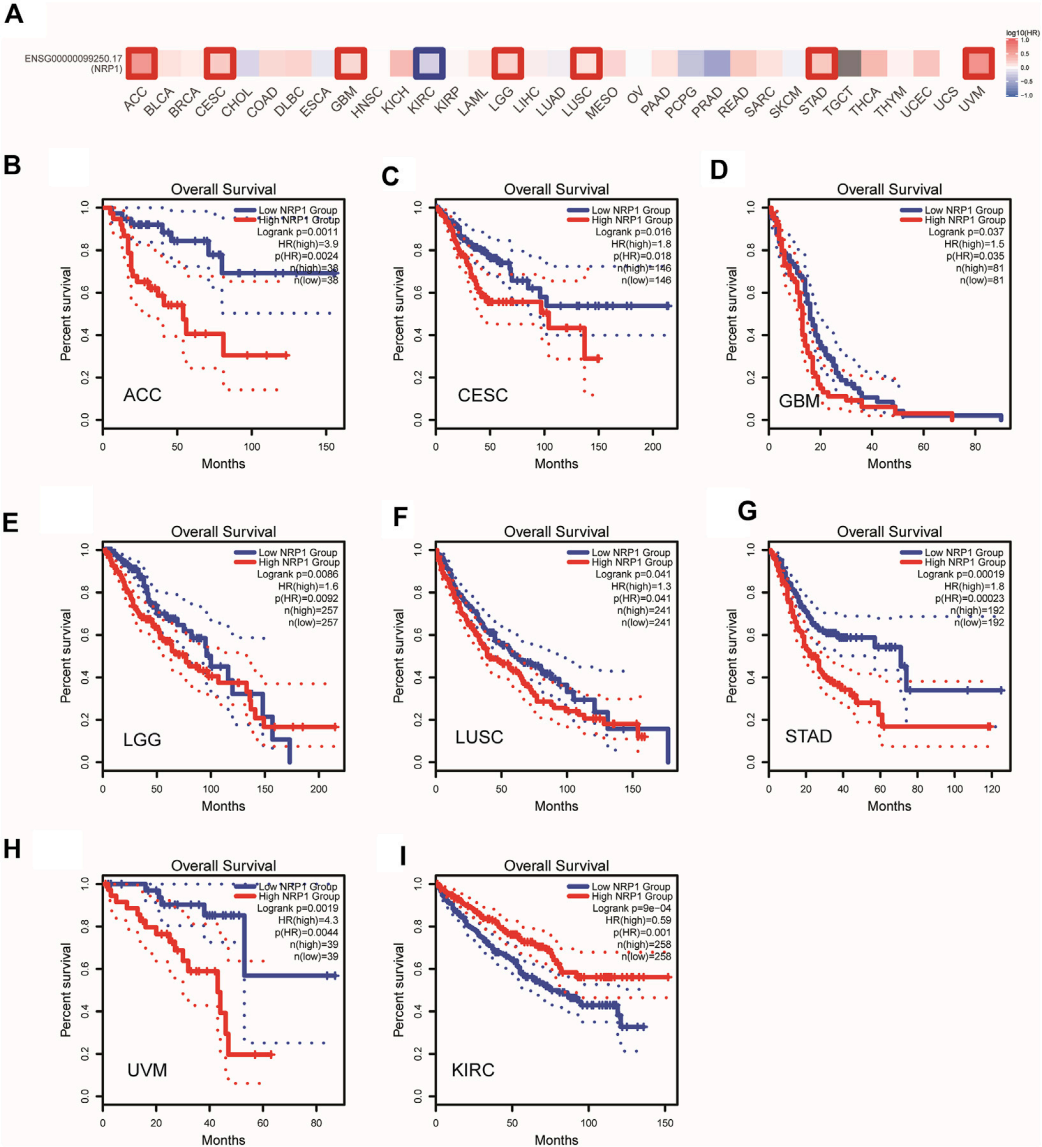
A pan-cancer correlation analysis between NRP1 expression and OS **(A)** An overview of correlation between NRP1 expression and OS in multiple cancer types. The red and blue blocks denoted higher and lower risks, respectively. **(B–I)** Kaplan-Meier plots of OS in the low NRP1 group and the high NRP1 group in ACC, CESC, GBM, LGG, LUSC, STAD, UVM, and KIRC, respectively. Dotted lines represent 95% confidence interval (CI).

We next did a pan-cancer analysis between NRP1 (ENSG00000099250.17) and OS of patients with GEPIA2. Cancer patients were divided into high- and low-NRP1 group according to median NRP1 expression level. An overview of the result was shown in Figure 4A. We found that NRP1 was negatively correlated with OS in seven cancer types, including adenoid cystic carcinoma (ACC) (*p* = 0.0011) (Figure 4B), cervical cancer (CESC) (*p* = 0.016) (Figure 4C), glioblastoma multiforme (GBM) (*p* = 0.037) (Figure 4D), low-grade glioma (LGG) (*p* = 0.0086) (Figure 4E), LUSC (*p* = 0.041) (Figure 4F), STAD (*p* = 0.00019) (Figure 4G), and uveal melanoma (UVM) (*p* = 0.0019) (Figure 4H). However, NRP1 was significantly positively correlated with OS in KIRC (P = 9e-04) (Figure 4I). A previous study also found that NRP1 expression was related with improved survival in renal cell carcinoma (Morin et al., 2020). Therefore, NRP1 functioned as an unfavorable prognostic marker in most cancer types.

### NRP1 expression correlated with immune environment in pan-cancers

**FIGURE 5:**
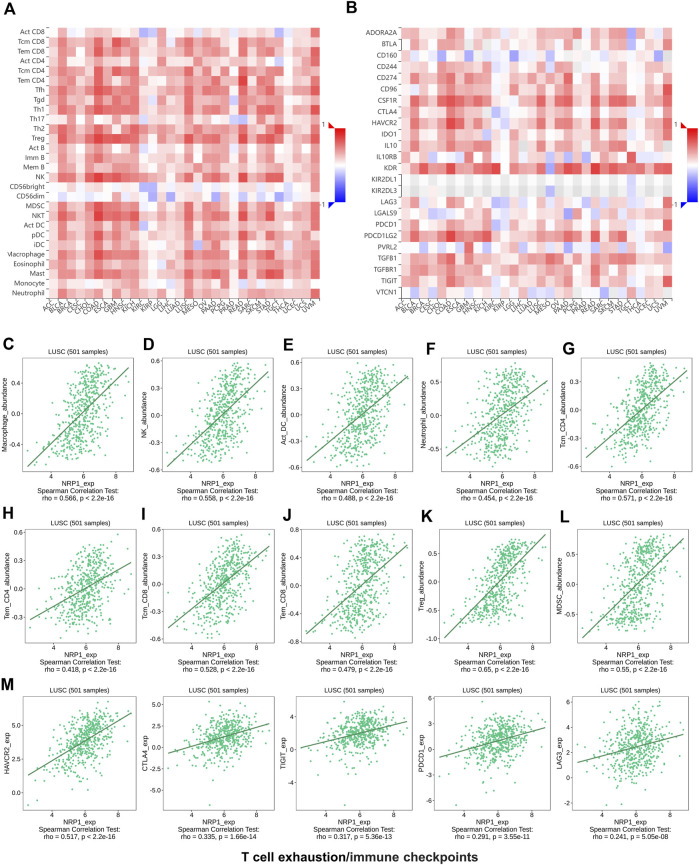
The relationship analysis between NRP1 expression and tumor immune environment using the TISIDB database **(A)** The relation between NRP1 expression and the abundance of different kinds of TILs in various cancers. **(B)** The relation between NRP1 expression and the expression of various immunoinhibitory markers across cancers **(C-L)** The correlation between NRP1 expression and the abundance of macrophages, NK cells, activated DCs, neutrophils, Tcm CD4^+^ cells, Tem CD4^+^ cells, Tcm CD8^+^ cells, Tem CD8^+^ cells, Tregs, and MDSCs in LUSC, respectively. **(M)** The correlation between NRP1 expression and the expression of HAVCR2, CTLA4, TIGIT, PDCD1, and LAG3 in LUSC, respectively.

As immune disorder plays an important role in both oncogenesis and virus infection, the correlation between NRP1 expression and immune infiltration levels was further investigated. Analysis using the TISIDB database showed that NRP1 expression was positively related with abundance of tumor infiltrating lymphocytes (TILs) (Figure 5A) and expression of immunoinhibitory markers (Figure 5B) in nearly all types of cancer, indicating that NRP1 expression could be an independent predictor of immune cell infiltration and response to immune checkpoint inhibitors (ICIs). We further investigated the correlation between NRP1 expression and cells or molecules related to immune in the TCGA-LUSC dataset containing 501 samples. Correlation analysis between NRP1 and TILs revealed that NRP1 expression was significantly positively correlated with the abundance of various immune cells including macrophages (rho = 0.566, *p* < 2.2e-16) (Figure 5C), NK cells (rho = 0.558, *p* < 2.2e-16) (Figure 5D), activated DCs (rho = 0.488, *p* < 2.2e-16) (Figure 5E), neutrophils (rho = 0.454, *p* < 2.2e-16) (Figure 5F), central memory (Tcm) CD4^+^ cells (rho = 0.571, *p* < 2.2e-16) (Figure 5G), effector memory (Tem) CD4^+^ cells (rho = 0.418, *p* < 2.2e-16) (Figure 5H), central memory (Tcm) CD8^+^ cells (rho = 0.528, *p* < 2.2e-16) (Figure 5I), effector memory (Tem) CD8^+^ cells (rho = 0.479, *p* < 2.2e-16) (Figure 5J), regulatory T cells (Treg) (rho = 0.65, *p* < 2.2e-16) (Figure 5K), and myeloid-derived suppressor cells (MDSCs) (rho = 0.55, *p* < 2.2e-16) (Figure 5L). Immune estimation using TIMER2.0 also revealed consistent results. NRP1 expression was negatively correlated with tumor purity (cor = -0.325, *p* = 3.27e-13), while positively related with B cells (partial.cor = 0.092, *p* = 4.53e-02), CD8^+^ cells (partial.cor = 0.32, *p* = 8.89e-13), CD4^+^ cells (partial.cor = 0.343, *p* = 1.56e-14), macrophages (partial.cor = 0.525, *p* = 3.59e-35), neutrophils (partial.cor = 0.474, *p* = 5.34e-28), and DCs (partial.cor = 0.533, *p* = 3.43e-36) in LUSC tumors (Supplementary Figure S1A). In addition, NRP1 expression was also significantly positively associated with several immunoinhibitory markers including HAVCR2 (rho = 0.517, *p* < 2.2e-16), CTLA4 (rho = 0.335, *p* < 1.66e-14), TIGIT (rho = 0.317, *p* < 5.36e-13), PDCD1 (rho = 0.291, *p* < 3.55e-11), and LAG3 (rho = 0.241, *p* < 5.05e-08) (Figure 5M) in LUSC tumors. These results could be validated in TIMER2.0 (Supplementary Figure S13B). Above findings indicated that NRP1 might participate in remodeling the TME of LUSC.

**FIGURE 6:**
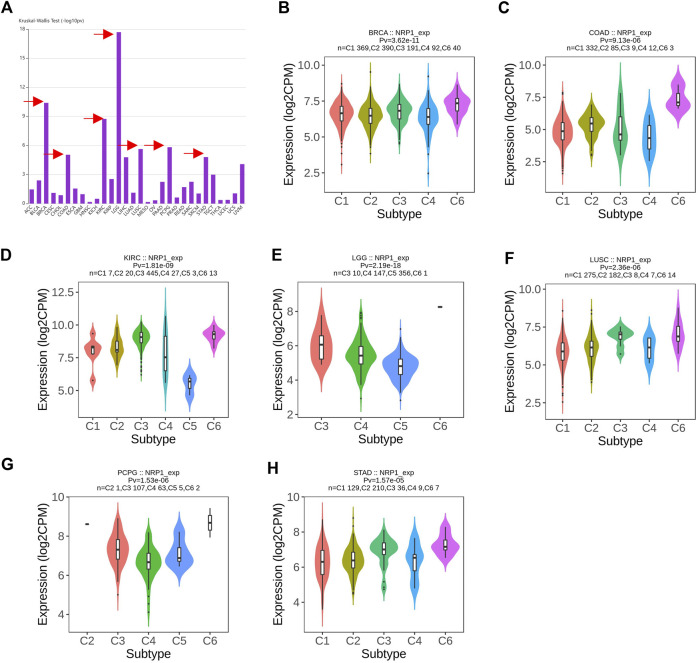
A pan-cancer landscape of NRP1 expression in different immune subtypes **(A)** Differential analysis of NRP1 expression among the six immune subtypes in different cancer types. Arrows indicated the top seven cancer types differentially expressing NRP1 among the six immune subtypes. **(B–H)** Violin plots showing NRP1 expression in the six immune subtypes in LGG, BRCA, KIRC, PCPG, LUSC, COAD, and STAD, respectively.

Analysis in TIMER2.0 revealed that NRP1 was significantly differentially expressed across the six immune subtypes in LGG (*p* = 2.19e-18), BRCA (*p* = 3.62e-11), KIRC (*p* = 1.81e-09), pheochromocytoma/paraganglioma (PCPG) (*p* = 1.53e-06), LUSC (*p* = 2.36e-06), COAD (*p* = 9.13e-06), and STAD (*p* = 1.57e-05) (Figure 6A). We did a further investigation and found that NRP1 was highly expressed in the TGF-β dominant immune subtype in all of these seven cancer types (Figures 6B–H), indicating that the TGF-β dominant immune subtype might be more susceptible to SARS-CoV-2 infection than other immune subtypes among LGG, BRCA, KIRC, PCPG, LUSC, COAD, and STAD patients.

Chemokines and their receptors influence tumorigenesis and metastases (Balkwill, 2004). The chemokine/chemokine-receptor system was also involved in the cytokine storm related to SARS-CoV-2 infection (Coperchini et al., 2020). Based on these we also investigated the relationship between NRP1 expression and chemokine/chemokine-receptor at a pan-cancer level in TISIDB. The result showed that both chemokines (Supplementary Figure S14A) and chemokine receptors (Supplementary Figure S14B) were positively correlated with the expression level of NRP1 in most cancer types, indicating that NRP1 could be a reliable predictor of chemokine storm caused by SARS-CoV-2 infection in cancer patients, and they might benefit from antibody cocktails targeting both NRP1 and chemokines/chemokine receptors.

## Discussion

The outbreak of COVID-19 worldwide has been a major challenge for global public health, so figuring out susceptible population to COVID-19 and predicting prognosis is of great importance from the perspective of epidemiology. Previous studies mainly focused on ACE2, a widely accepted receptor of SARS-CoV-2. However, understanding other potential receptors of SARS-CoV-2 may also be helpful in offering novel insights into the pathogenesis and treatment of SARS-CoV-2. In this article, we compared the expression level of NRP1 in different tissues and organs among both healthy people, COVID-19 patients and cancer patients. We also compared the expression of NRP1 with that of ACE2 directly. Besides, we did a pan-cancer analysis of the relationship between NRP1 expression and OS, NRP1 mutation, and TME.

We found that NRP1 was highly conserved in different species. Experimental studies have indeed shown that a small number of animals are susceptible to infection with SARS-CoV-2 and can transmit the virus (Shi et al., 2020; Sit et al., 2020).

Our study revealed that NRP1 was highly expressed in female reproductive system, respiratory system, and urinary system. In accordance with the expression profile observed in normal tissues, NRP1 was also highly expressed in ovarian cancer, colorectal cancer, and kidney cancer. This indicated that NRP1 mainly expressed in tissues communicating the outer environment, which was similar with that of ACE2 (Hamming et al., 2004). It is worth noticing that NRP1 was highly expressed in placenta. Researchers have already shown that SARS-CoV-2 localized predominantly to syncytiotrophoblast cells at the materno-fetal interface of the placenta in a pregnant woman with COVID-19 (Hosier et al., 2020), but there was no direct evidence of vertical transmission (Edlow et al., 2020). Interestingly, compared with the mRNA level, the protein level of NRP1 was higher in respiratory system and congestive system, indicating that post-transcriptional modifications of NRP1 mRNA may exist in different tissues. At the single-cell level, NRP1 was highly expressed in macrophages and endothelial cells in both normal tissues and cancerous. Pulmonary macrophages are at the heart of the airway innate immunity (Byrne et al., 2015), so macrophage dysfunction caused by the infection of SARS-CoV-2 makes COVID-19 patients vulnerable to bacterial infection (Langford et al., 2020). Previous studies had already verified macrophage as a therapeutic target in COVID-19 cancer patients (Sica et al., 2020). Besides, evidence showed that the virus could infect endothelial cells and cause diffuse endothelial inflammation (Varga et al., 2020). The extremely low expression of ACE2 in lung could hardly explain the severity of pulmonary distress syndrome in SARS-CoV-2-infected patients. Comparison between ACE2 and NRP1 revealed that expression level of NRP1 was higher than that of ACE2 in most tumor and normal tissues (including lung tissues and plasma). However, different from ACE2, NRP1 was not highly expressed in gastrointestinal tract. Our finding added evidence to the idea that ACE2 was not the only receptor involved in the infection process of SARS-CoV-2, NRP1 may also contribute to virus infection and virulence. Besides, we found that NRP1 was significantly higher in lung tissues from COVID-19 patients than non-COVID-19 people at both bulk and single-cell level, which was consistent with previous studies (Cantuti-Castelvetri et al., 2020; Daly et al., 2020). Thus, we speculated that NRP1 was a potential valuable biomarker in predicting susceptibility to SARS-CoV-2 infection and targeting NRP1 in treating COVID-19 patients might achieve ideal clinical results.

Pan-cancer analysis revealed that the isoform NRP1-202 might be involved in both tumorigenesis and SARS-CoV-2 infection processes. We found that expression level and mutation rate of NRP1 depended on cancer types. However, in most cancer types, NRP1 functioned as a potential oncogene, which was consistent with previous studies (Vescarelli et al., 2020; Liu et al., 2021; Song et al., 2021; Yin et al., 2021). This indicated that cancer patients with poor prognosis were more susceptible to SARS-CoV-2 infection than healthy people. Relationship between NRP1 and TME was also intricate. Components of TILs were complicated and they could be either tumor-promoting or tumor-suppressing (Paijens et al., 2021). Our results indicated that NRP1 was a double-edge sword for TME. The TGF-β dominant immune subtype was characterized by a high lymphocytic infiltration with an even distribution of Type I and Type II T cells (Thorsson et al., 2018). It is worth noticing that the TGF-β dominant immune subtype of cancers related to respiratory system, digestive system, and female reproductive system was more susceptible to SARS-CoV-2 than other immune subtypes. Susceptible patients might benefit from precision medicine combining NRP1 inhibitors and immunotherapy.

In most cancer types expression of NRP1 was positively related with expression of immunoinhibitory markers and chemokines/chemokine-receptors. ICIs have gradually became an irreplaceable role in cancer therapeutics in recent years (Darvin et al., 2018), and inhibiting the chemokine system has also been emerging as a potential target for immunotherapy in cancer (Poeta et al., 2019). Cancer patients with high expression level of NRP1 were presumed to be vulnerable to the infection of SARS-CoV-2, and they were more likely to experience cytokine storm. However, these patients might be potential responders to the combination therapy of ICIs, chemokine inhibitors, and NRP1 inhibitors, which could be beneficial to suppressing tumor development, and controlling symptoms of COVID-19 at the same time. This treatment might be efficient in reducing viral load as well as relieving severe symptoms of COVID-19 patients. In fact, the PD-1 antibody nivolumab is currently being investigated in a phase Ⅱ clinical trial (NCT04356508). Clinical trials evaluating the effect of anti-PD-1 in COVID-19 patients complicated with cancer are also needed to reduce the mortality rate of such patients.

Our research identified NRP1 as a valuable marker in predicting vulnerability to SARS-CoV-2 infection among normal people, however, the susceptibility to SARS-CoV-2 in cancer patients merits further investigation. The COVID-19 and Cancer Consortium (CCC19) is an international consortium which collects data on patients with cancer and COVID-19 (Rivera et al., 2020). Studies had already proved that patients with cancer were at increased risk of severe COVID-19 outcomes (Zhang et al., 2021), but a cohort study showed that cancer type was not associated with mortality (Kuderer et al., 2020). However, this conclusion might be biased by the limited follow-up time. Besides, more controlled studies comparing clinical and laboratory features between cancer and non-cancer patients are urgently needed.

In conclusion, our study figured out susceptible population to SARS-CoV-2 based on NRP1 expression. NRP1 could be a valuable informative marker in predicting susceptibility to SARS-CoV-2 and the severity of COVID-19 patients, and it had a great potential as a therapeutic target especially in cancer patients combined with COVID-19.

## Data Availability

The original contributions presented in the study are included in the article/Supplementary Material further inquiries can be directed to the corresponding authors.
