# Supplementary material for: Characterization of the SARS-CoV-2 co-receptor NRP1 expression profiles in healthy people and cancer patients: Implication for susceptibility to COVID-19 disease and potential therapeutic strategy
Source: Front Genet. 2022 Oct 19;13:995736. doi: 10.3389/fgene.2022.995736 (PMC9627153; doi:10.3389/fgene.2022.995736)
Supplement: Supplementary file 1 [file Image2.PDF]

Supplementary material 2

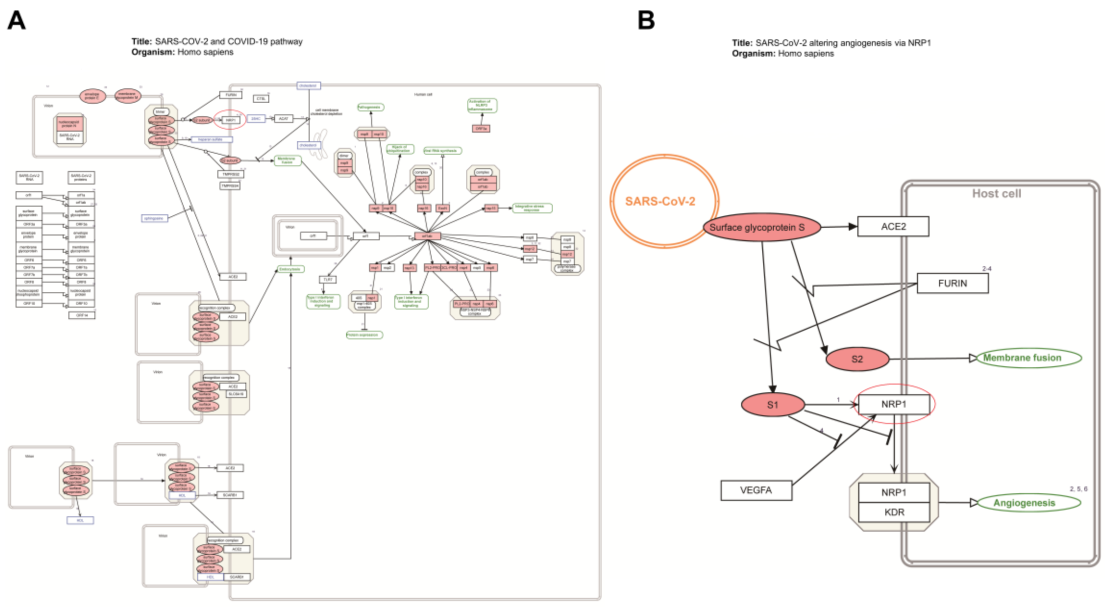

Figure S1. Role of NRP1 in SARS-CoV-2 infection process. **(A)** SARS-CoV-2 and COVID-19 pathway. **(B)** SARS-CoV-2 altering angiogenesis via NRP1. Red circles highlighted NRP1 on the cell membrane.

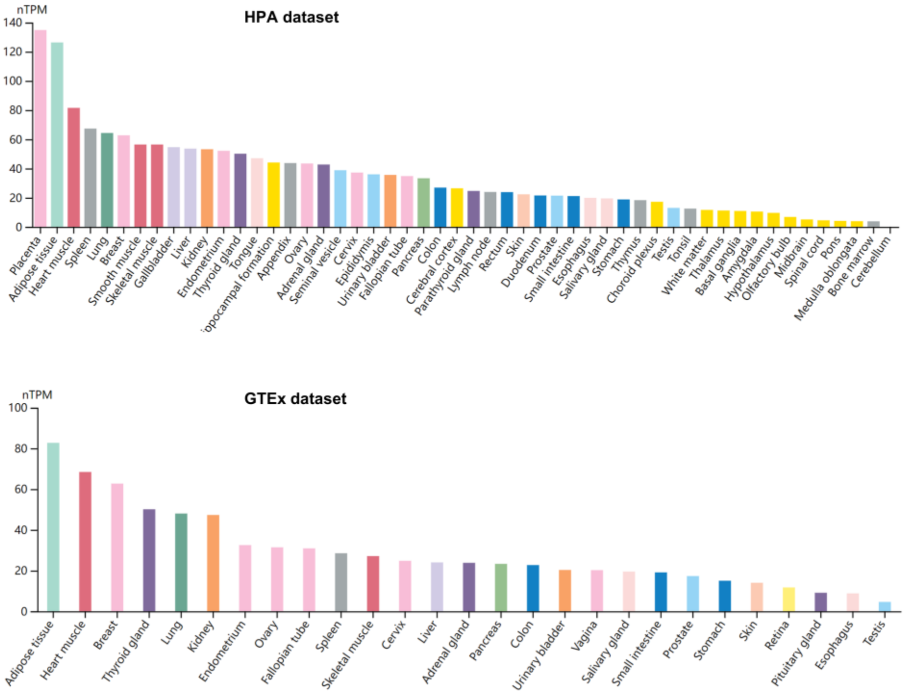

Figure S2. NRP1 expression in normal human tissues. **(A**) The mRNA expression profile of NRP1 in HPA dataset ranked by expression levels. **(B)** The mRNA expression profile of NRP1 in GTEx dataset ranked by expression levels.

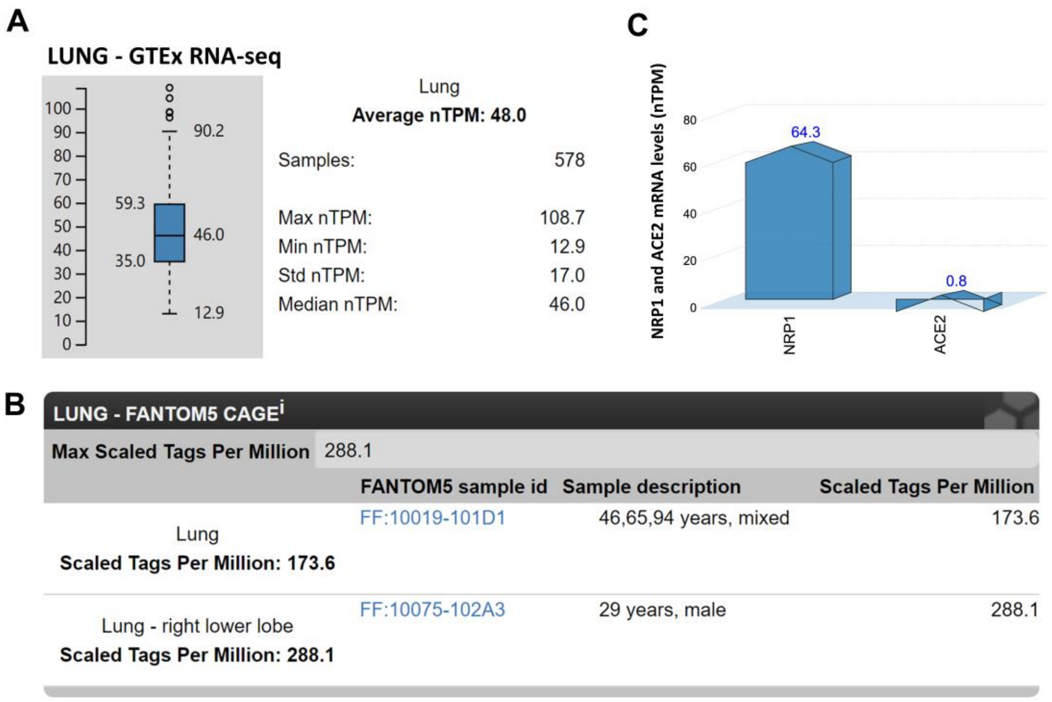

Figure S3. NRP1 expression in healthy human lungs. **(A)** NRP1 mRNA expression level of lung tissues in GTEx dataset. **(B)** NRP1 mRNA expression level of lung tissues in FANTOM5 dataset. **(C)** Comparison between mRNA expression levels of ACE2 and NRP1 in normal lung tissues from the consensus dataset.

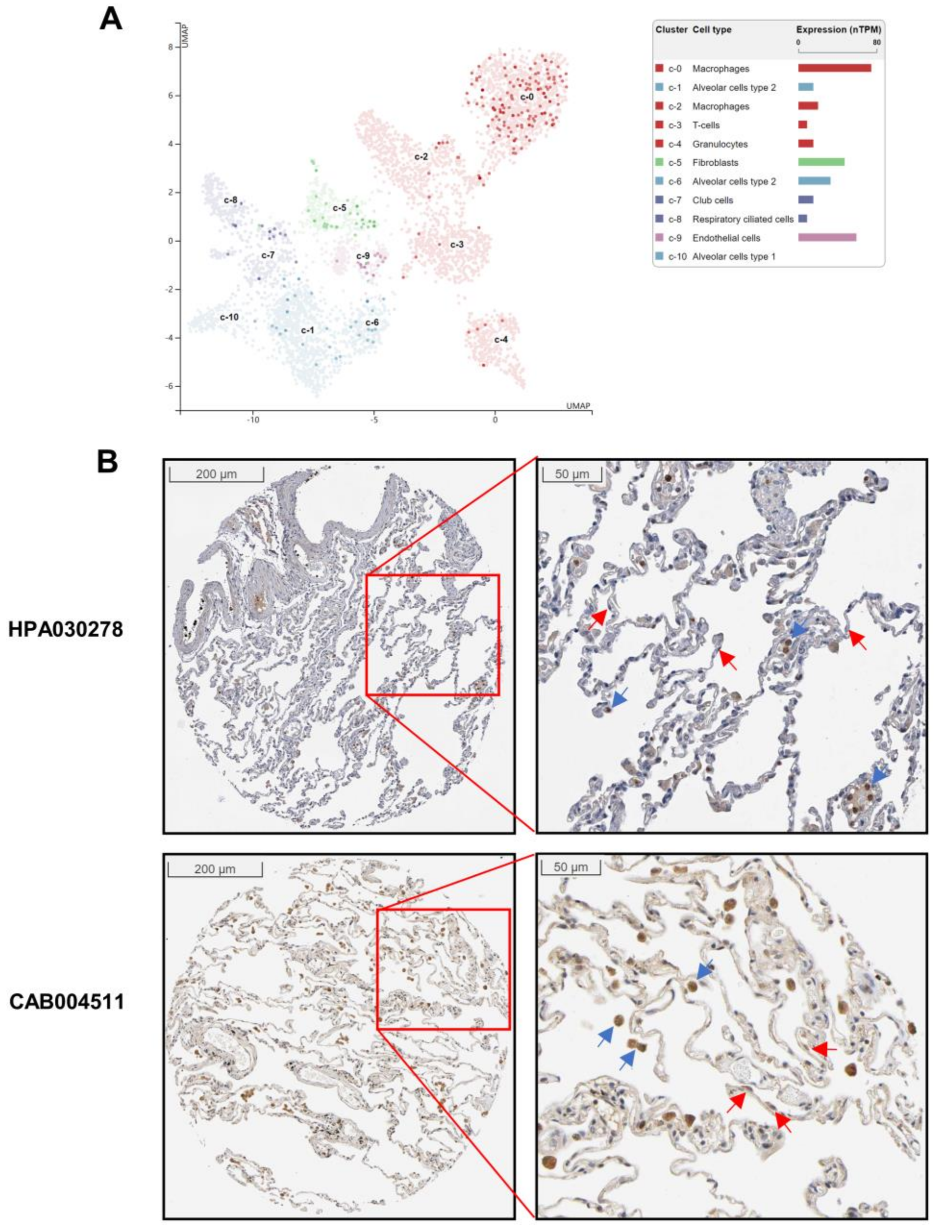

Figure S4. NRP1 expression in healthy human lungs. **(A)** UMAP plot showing NRP1 mRNA expression in different cell types of normal lung tissues. **(B)** Representative IHC images showing cellular localization of NRP1 in normal lung tissues. The upper IHC images were normal lung tissues of a 49-year-old female (Patient id: 2268) stained with NRP1 antibody HPA030278. The lower IHC images were normal lung tissues of a 69-year-old male (Patient id: 2373) stained with NRP1 antibody CAB004511. Enlarged images were presented in the right panel. Scale bars were 200

μm and 50 μm respectively. Arrows in blue indicated representative positive results for macrophages, and arrows in red indicated representative positive results for alveolar epithelial cells.

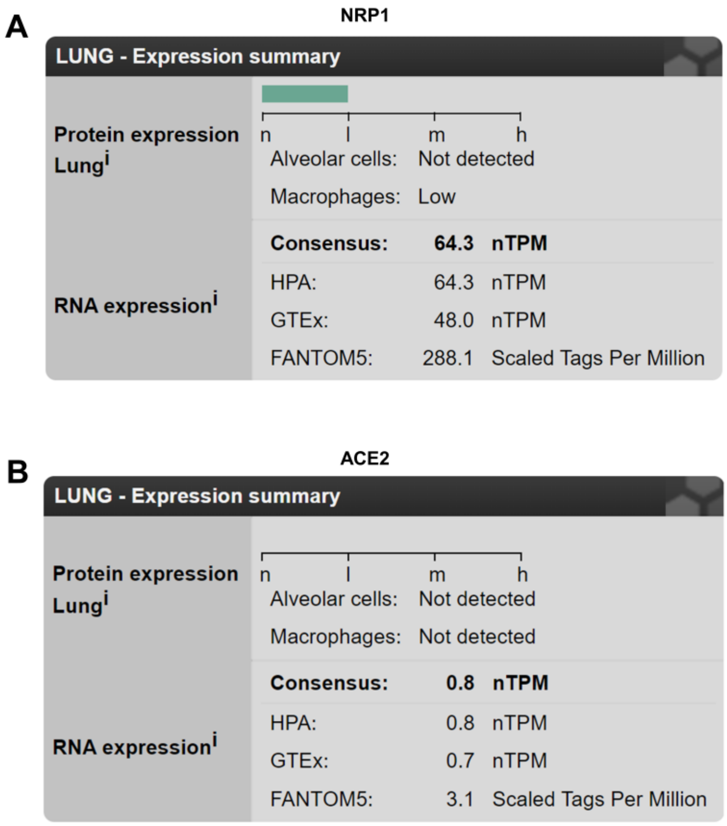

Figure S5. NRP1 and ACE2 mRNA expression levels in healthy human lungs from various datasets. **(A)** Summary of NRP1 mRNA expression level of lung tissues in different datasets. **(B)** Summary of ACE2 mRNA expression level of lung tissues in different datasets.

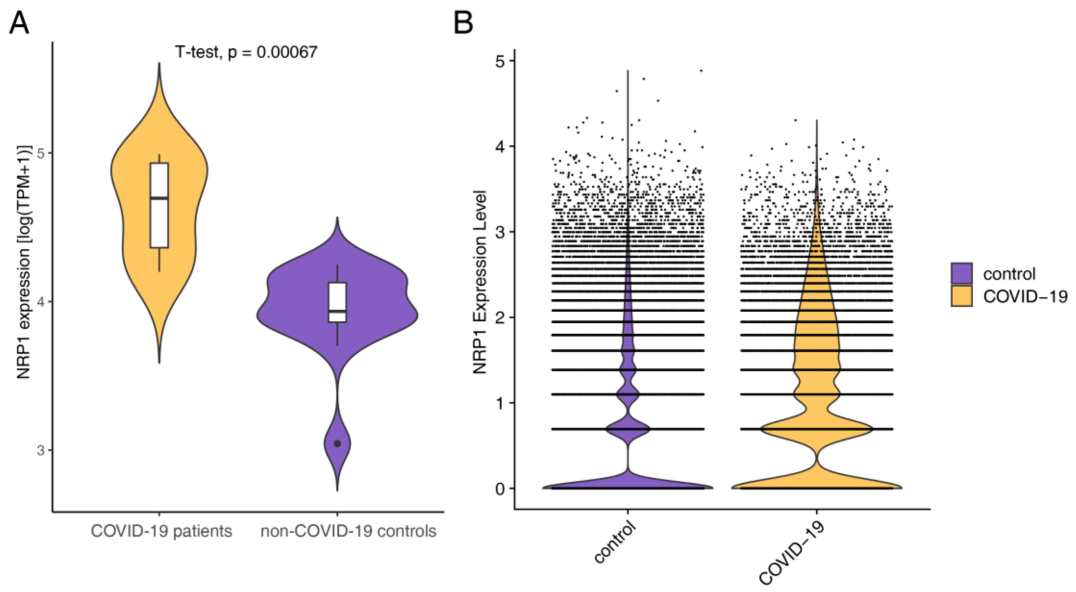

Figure S6. Comparison of the NRP1 expression level between COVID-19 patients and non-COVID-19 controls. **(A)** Violin plot showing the NRP1 expression at the bulk level. **(B)** Violin plot showing the NRP1 expression at the single-cell level.

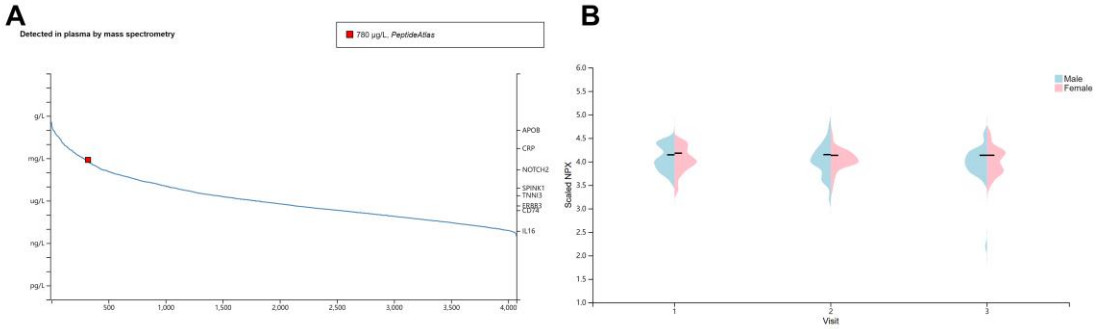

Figure S7. NRP1 protein concentration in plasma. **(A)** The protein concentration of NRP1 detected in plasma by mass spectrometry. **(B)** The difference of plasma NRP1 protein concentration between male and female detected by PEA.

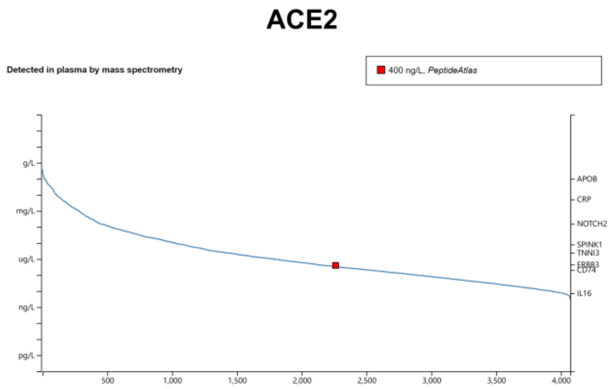

Figure S8. The protein concentration of ACE2 detected in plasma by mass spectrometry.

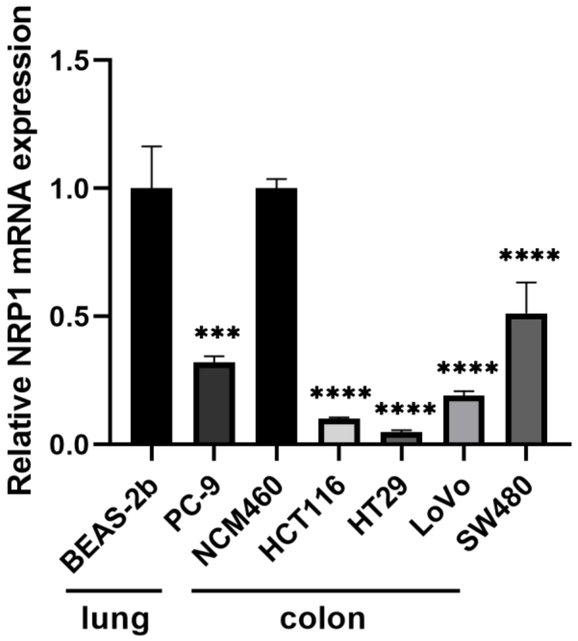

Figure S9. Relative NRP1 expression level in BEAS-2b, PC-9, NCM460, HCT116, HT29, LoVo, and SW480 detected by qRT-PCR. *** represents P < 0.01, **** represents P < 0.0001.

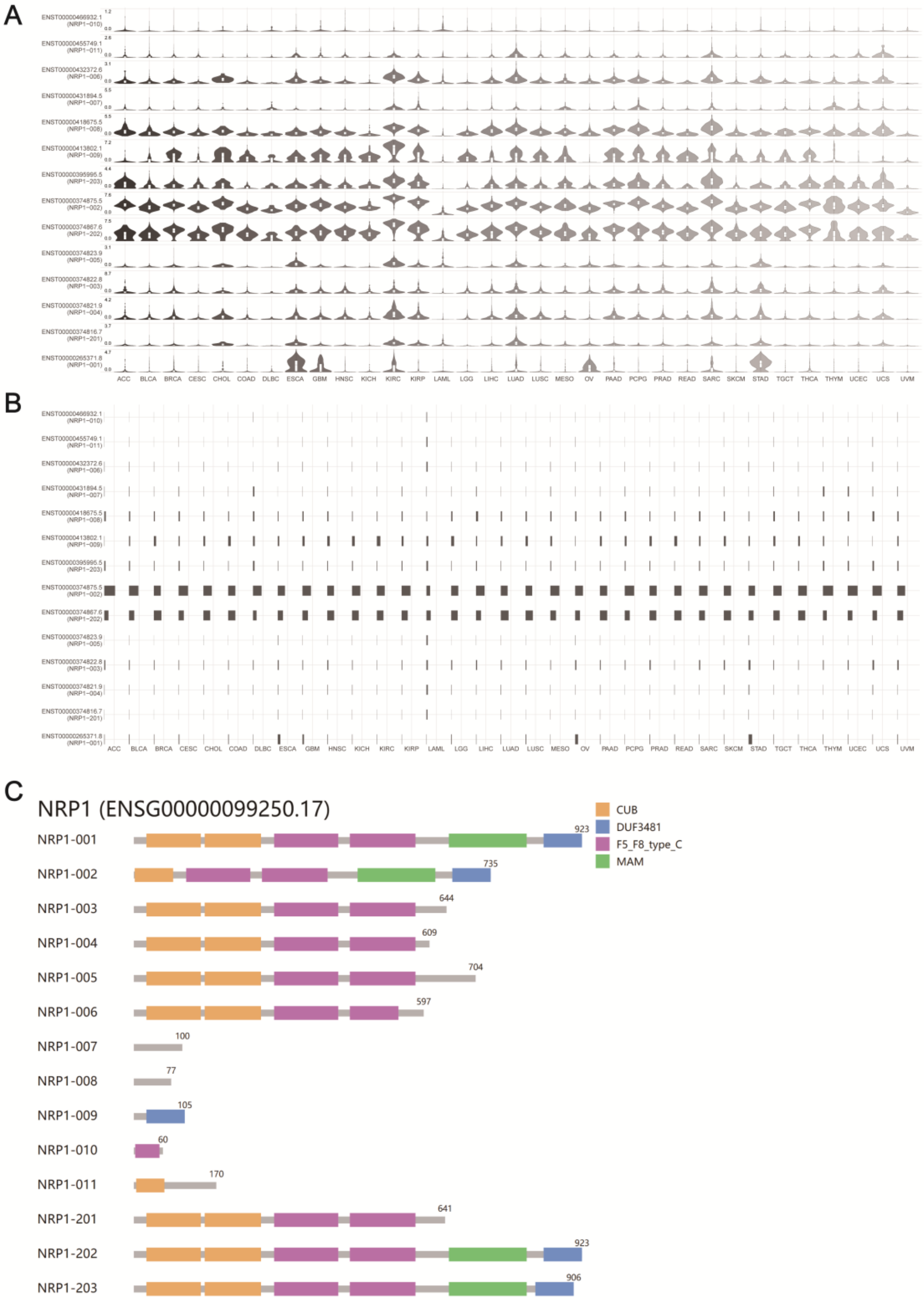

Figure S10. The usage and structure of NRP1 isoforms in different types of cancers. **(A)** Violin plot showing the expression distribution of NRP1 isoforms. **(B)** Bar plot showing the usage profile of NRP1 isoforms. **(C)** Isoform structures of NRP1.

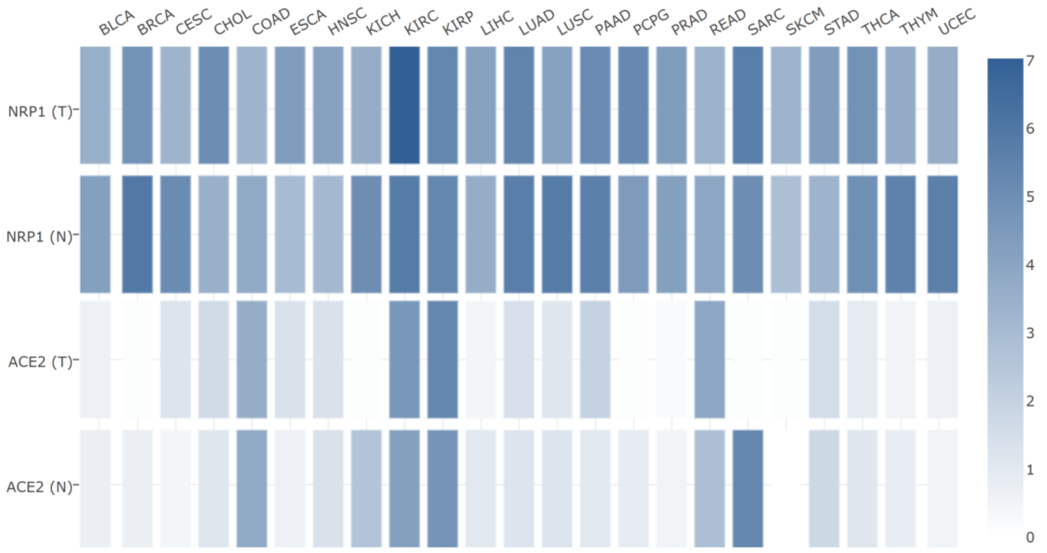

Figure S11. Heatmap showing expression comparisons between NRP1 and ACE2 in different tumors and matched normal samples in the TCGA dataset. “T” represents tumor tissues and “N” normal tissues. The density of color in each block represented the median expression level of a certain gene in a given tissue, normalized by the maximum median expression level across all blocks.

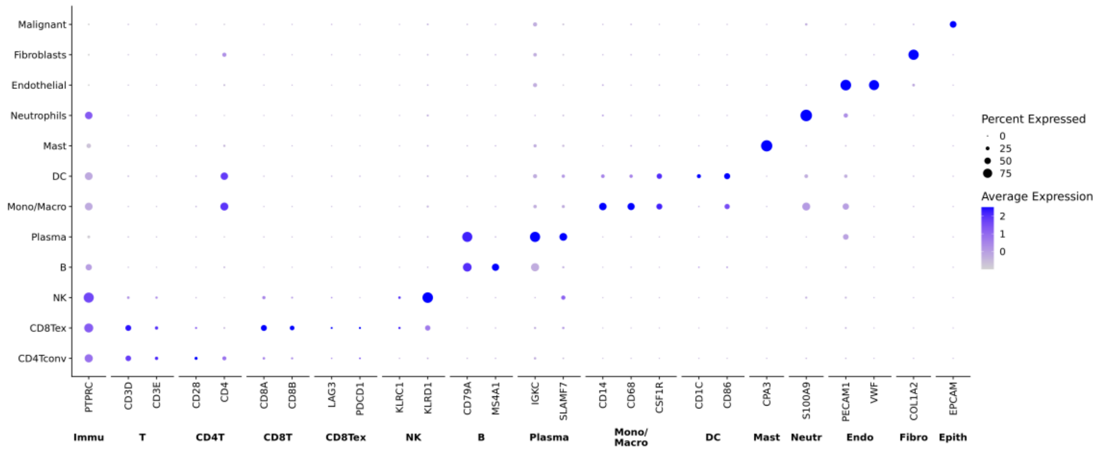

Figure S12. Dot plot showing the expression level of marker genes for all the annotated cell types in the NSCLC_GSE127465 dataset.

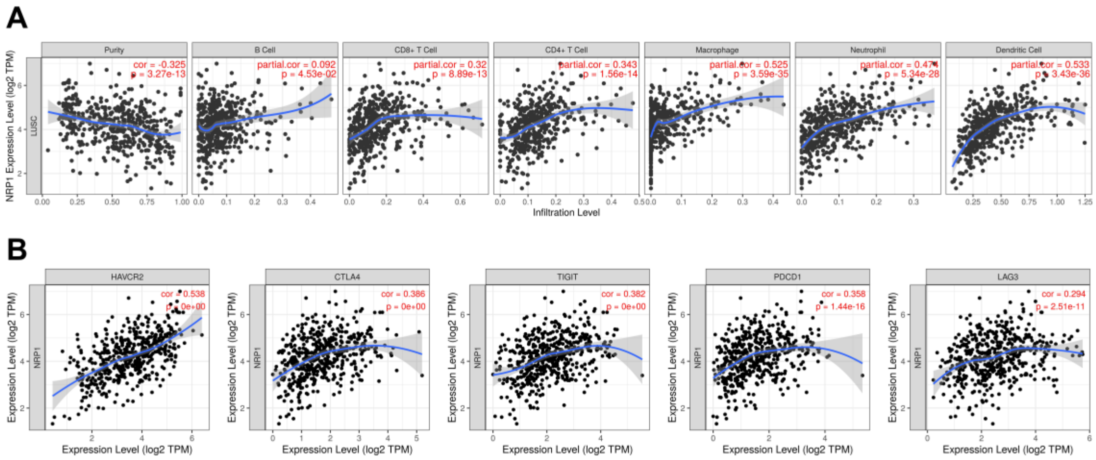

Figure S13. The relationship analysis between NRP1 expression and tumor immune environment in LUSC using the TIMER2.0 database. **(A)** The correlation between NRP1 expression and tumor purity, the abundance of B cells, $CD8^+$ cells, $CD4^+$ cells, macrophages, neutrophils, and DCs in LUSC tumors. (B) The correlation between NRP1 expression and the expression of HAVCR2, CTLA4, TIGIT, PDCD1, and LAG3 in LUSC.

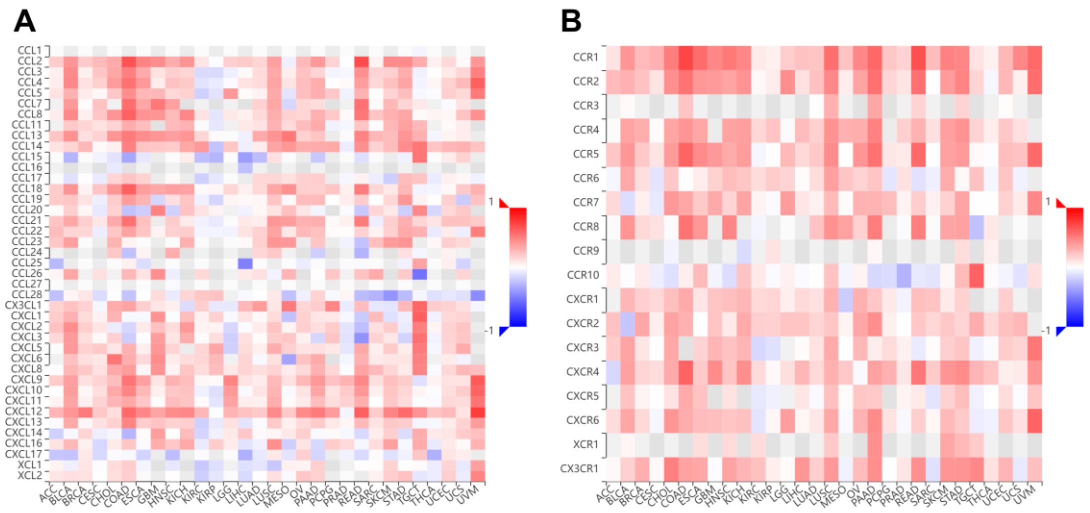

Figure S14. The relationship analysis between NRP1 expression and tumor immune environment using the TISIDB database. **(A)** The relation between NRP1 expression and the abundance of different kinds of chemokines in various cancers. **(B)** The relation between NRP1 expression and the expression of various chemokine receptors across cancers.
