# Supplementary material for: Characterization of the SARS-CoV-2 co-receptor NRP1 expression profiles in healthy people and cancer patients: Implication for susceptibility to COVID-19 disease and potential therapeutic strategy
Source: Front Genet. 2022 Oct 19;13:995736. doi: 10.3389/fgene.2022.995736 (PMC9627153; doi:10.3389/fgene.2022.995736)
Supplement: Supplementary file 2 [file Image1.PDF]

Results colour-coded for amino acid conservation

The current colourscheme of the alignment is for **amino acid conservation**.

The conservation scoring is performed by PRALINE. The scoring scheme works from 0 for the least conserved alignment position, up to 10 for the most conserved alignment position. The colour assignments are:

Unconserved 0 1 2 3 4 5 6 7 8 9 10 **Conserved**

```
                 .........10 .........20 .........30 .........40 .........50
NP_003864_5_neu  MERGLPLLCA VLALVLAP-- AGAFRNDKCG DTIKIESPGY LTSPGYPHSY
XP_001143690_1_  MESGLPLLCA VLALVLAP-- AGAFRNDKCG DTIKIESPGY LTSPGYPHSY
NP_001252745_1_  MEKGLPLLCA ALALALAP-- AGAFRNDKCG DTIKIESPGY LTSPGYPHSY
NP_001192589_1_  MEKGLQLLCA ALALVLGA-- AGAFRNDKCG DTIKIESPGY LTSPGYPHSY
XP_005617003_1_  MERGPPLLSA ALAVAVAVAL AGAFRNDKCG DTIKIESPGY LTSPGYPHSY
NP_032763_2_neu  MERGLPLLCA TLALALAL-- AGAFRSDKCG GTIKIENPGY LTSPGYPHSY
NP_659566_1_neu  MERGLPLLCA TLALALAL-- AGAFRSDKCG GTIKIENPGY LTSPGYPHSY
NP_990113_1_neu  MDWGLFLHCA ALTFTL---- SRALRSDKCG DTIKILSPGY LTSPGYPQSY
NP_001093692_1_  --MLLRLLSC WLLCSLRS-- SWASRNDKCG DTIKITNPSY LTSTGYPHSY
NP_852474_2_neu  MHCGLVLILF TGIFLI---- VSALKNDKCG DNIRITSANY LTSPGYPVSY
Consistency      864884*767 5866584200 76*697**** 78*9*6787* ***8***7**

                 .........60 .........70 .........80 .........90 ........100
NP_003864_5_neu  HPSEKCEWLI QAPDPYQRIM INFNPHFDLE DRDCKYDYVE VFDGENENGH
XP_001143690_1_  HPSEKCEWLI QAPDPYQRIM INFNPHFDLE DRDCKYDYVE VFDGENENGH
NP_001252745_1_  HPSEKCEWLI QAPDPYQRIM INFNPHFDLE DRDCKYDYVE VFDGENENGR
NP_001192589_1_  HPSEKCEWLI QAPDPYQRIM INFNPHFDLE DRDCKYDYVE VIDGENESGR
XP_005617003_1_  HPSEKCEWLI QAPDPYQRIM INFNPHFDLE DRDCKYDYVE VIDGENENGR
NP_032763_2_neu  HPSEKCEWLI QAPEPYQRIM INFNPHFDLE DRDCKYDYVE VIDGENEGGR
NP_659566_1_neu  HPSEKCEWLI QAPEPYQRIM INFNPHFDLE DRDCKYDYVE VIDGENEGGR
NP_990113_1_neu  HPSQKCEWLI QAPEPYQRIM INFNPHFDLE DRDCKYDYVE VIDGDNAEGR
NP_001093692_1_  LPSQRCEWLI QAPEQYQRIM INFNPHFDLE DRECKYDYVE VIDGDNASGQ
NP_852474_2_neu  YPSQKCIWVI TAPGPNQRIL INFNPHFDLE DRECKYDYVE VRDGVDENGQ
Consistency      7**89*8*9* 8**688***9 ********** **8******* *6**7975*6

                 ........110 ........120 ........130 ........140 ........150
NP_003864_5_neu  FRGKFCGKIA PPPVVSSGPF LFIKFVSDYE THGAGFSIRY EIFKRGPECS
XP_001143690_1_  FRGKFCGKIA PPPVVSSGPF LFIKFVSDYE THGAGFSIRY EIFKRGPECS
NP_001252745_1_  LWGKFCGKIA PPPVVSSGQF LFIKFVSDYE THGAGFSIRY EIFKRGPECS
NP_001192589_1_  VLGKFCGKIA PSSFVSSGPF LFIKFVSDYE THGAGFSIRY EIFKKGPECS
XP_005617003_1_  PWGKFCGKIA PSPVVSSGPF LFIKFVSDYE THGAGFSIRY EIFKRGPECS
NP_032763_2_neu  LWGKFCGKIA PSPVVSSGPF LFIKFVSDYE THGAGFSIRY EIFKRGPECS
NP_659566_1_neu  LWGKFCGKIA PSPVVSSGPF LFIKFVSDYE THGAGFSIRY EIFKRGPECS
NP_990113_1_neu  LWGKYCGKIA PPPLVSSGPY LFIKFVSDYE THGAGFSIRY EVFKRGPECS
NP_001093692_1_  LLGKFCGKIA PSPLVSTGPS IFIRFVSDYE THGAGFSIRY EVFKTGPECS
NP_852474_2_neu  LVGKYCGKIA PSPVVSSGNQ LFIKFVSDYE THGAGFSIRY EIFKTGPECS
Consistency      53**8***** *687**9*76 9**9****** ********** *9**7*****

                 ........160 ........170 ........180 ........190 ........200
NP_003864_5_neu  QNYTTPSGVI KSPGFPEKYP NSLECTYIVF VPKMSEIILE FESFDLEPDS
XP_001143690_1_  QNYTTPSGVI KSPGFPEKYP NSLECTYIVF APKMSEIILE FESFDLEPDS
NP_001252745_1_  QNYTTPSGVI KSPGFPEKYP NSLECTYIVF APKMSEIILE FESFDLEPDS
NP_001192589_1_  QNYTMPSGII KSPGFPEKYP NSLECTYIIF APKMSEIILE FESFDLEPDS
XP_005617003_1_  QNYTAPSGVI KSPGFPEKYP NGLECTYIIF APKMSEIILE FESFDLELDS
NP_032763_2_neu  QNYTAPTGVI KSPGFPEKYP NSLECTYIIF APKMSEIILE FESFDLEQDS
NP_659566_1_neu  QNYTAPTGVI KSPGFPEKYP NSLECTYIIF APKMSEIILE FESFDLEQDS
NP_990113_1_neu  RNFTSSSGMI KSPGFPEKYP NSLECTYIIF APKMSEIILE FESFELEPDS
NP_001093692_1_  RNFTSSNGVI KSPKYPEKYP NALECTYIIF APKMLEIVLE FESFELEPDA
NP_852474_2_neu  RNFTSSSGVI KSPGFPEKYP NNLDCTFMIF APKMSEIVLE FESFELEPDT
Consistency      7*8*667*8* ***89***** *7*9**999* 9***8**9** ****8**6*8

                 ........210 ........220 ........230 ........240 ........250
NP_003864_5_neu  NPPGGMFCRY DRLEIWDGFP DVGPHIGRYC GQKTPGRIRS SSGILSMVFY
XP_001143690_1_  NPPGGMFCRY DRLEIWDGFP DVGPHIGRYC GQKTPGRIRS SSGILSMVFY
NP_001252745_1_  NPPGGMFCRY DRLEIWDGFP DVGPHIGRYC GQKTPGRIRS SSGILSMVFY
NP_001192589_1_  NPPGGMFCRY DRLEIWDGFP DVGPHIGRYC GQKTPGRILS SSGILSMLFY
XP_005617003_1_  NPPGGMFCRY DRLEIWDGFP DVGPHIGRYC GQKTPGRIRS SSGILSMVFY
NP_032763_2_neu  NPPGGMFCRY DRLEIWDGFP EVGPHIGRYC GQKTPGRIRS SSGVLSMVFY
NP_659566_1_neu  NPPGGVFCRY DRLEIWDGFP EVGPHIGRYC GQKTPGRIRS SSGILSMVFY
NP_990113_1_neu  NTPGGAFCRY DRLEIWDGFP DVGPHIGRYC GQNNPGRVRS STGILSMVFY
NP_001093692_1_  NAPVGQMCRY DWLGIWDGFP GVGPHIGRYC GQNTPGRVRS TTGILSMIFH
NP_852474_2_neu  QPPAGVFCRY DRLEIWDGFP GVGPYIGRYC GQNTPGRIIS YTGTLAMTIN
Consistency      87*7*68*** *8*8****** 6***9***** **78***97* 78*8*9*887
```

```
                . . . . . . . . . 260. . . . . . . . . 270. . . . . . . . . 280. . . . . . . . . 290. . . . . . . . . 300
NP_003864_5_neu TDSAIAKEGF SANYSVLQSS VSEDFKCMEA LGMESGEIHS DQITASSQYS
XP_001143690_1_ TDSAIAKEGF SANYSVLQSS VSEDFKCMEA LGMESGEVHS DQITASSQYS
NP_001252745_1_ TDSAIAKEGF SANYSVLQSS VSEDFKCMEA VGMESGEIHS DQITASSQYS
NP_001192589_1_ TDSAIAKEGF SANYSVLQSS VSEDFKCMEA LGMESGEIHS DQITASSQYS
XP_005617003_1_ TDSAIAKEGF SANYSVLQSS VSEDFKCMEA LGMESGEIHS DQITASSQYS
NP_032763_2_neu TDSAIAKEGF SANYSVLQSS ISEDFKCMEA LGMESGEIHS DQITASSQYG
NP_659566_1_neu TDSAIAKEGF SANYSVLQSS ISEDFKCMEA LGMESGEIHS DQITASSQYG
NP_990113_1_neu TDSAIAKEGF SANYSVSQSS VSEDFQCMEP LGMESGEIHS DQITVSSQYS
NP_001093692_1_ TDSAIAKEGF FANFSVVQSN TNEDFQCKEP LGMESGEIHF DQISVSSQYS
NP_852474_2_neu TDSAIAKEGF SANFTVLERT VPDDFDCTEP LGMETGEIHS DQIMASSQYS
Consistency     ********** 8**89*7988 879**7*7*6 9***9**9*8 ***78****8

                . . . . . . . . . 310. . . . . . . . . 320. . . . . . . . . 330. . . . . . . . . 340. . . . . . . . . 350
NP_003864_5_neu TNWSAERSRL NYPENGWTPG EDSYREWIQV DLGLLRFVTA VGTQGAISKE
XP_001143690_1_ TNWSAERSRL NYPENGWTPG EDSYREWIQV DLGLLRFVTA VGTQGAISKE
NP_001252745_1_ TNWSAERSRL NYPENGWTPG EDSYREWIQV DLGLLRFVTA VGTQGAISKE
NP_001192589_1_ TNWSAERSRL HYPENGWTPG EDSYREWIQV DLGLLRFVTA VGTQGAISKE
XP_005617003_1_ TNWSAERSRL NYPENGWTPG EDSYREWIQV DLGLLRFVTA VGTQGAISKE
NP_032763_2_neu TNWSVERSRL NYPENGWTPG EDSYKEWIQV DLGLLRFVTA VGTQGAISKE
NP_659566_1_neu TNWSVERSRL NYPENGWTPG EDSYREWIQV DLGLLRFVTA VGTQGAISKE
NP_990113_1_neu AIWSSERSRL NYPENGWTPG EDSVREWIQV DLGLLRFVSG IGTQGAISKE
NP_001093692_1_ MNWSAERSRL NYVENGWTPG EDTVKEWIQV DLENLRFVSG IGTQGAISKE
NP_852474_2_neu NSWSAERSRL NNPENGWTPL EDTNKEGIQV DLGFLRFVSA IGTQGAISQE
Consistency     67**7***** 888******8 **868*8*** **87****88 9*******9*

                . . . . . . . . . 360. . . . . . . . . 370. . . . . . . . . 380. . . . . . . . . 390. . . . . . . . . 400
NP_003864_5_neu TKKKYYVKTY KIDVSSNGED WITIKEGNKP VLFQGNTNPT DVVVAVFPKP
XP_001143690_1_ TKKKYYVKTY KIDVSSNGED WITIKEGNKP VLFQGNTNPT DVVVAVFPKP
NP_001252745_1_ TKKKYYVKTY KIDISSNGED WITIKEGNKP VLFQGNTNPT DVVVAVFPKP
NP_001192589_1_ TRKKYYVKTY RIDISSNGED WITIKEGNKP VIFHGNTNPT DVVIGVFPKP
XP_005617003_1_ TKKKYYVKTY RIDISSNGED WITIKEGNKP VIFQGNTNPT DVVFAVFPKP
NP_032763_2_neu TKKKYYVKTY RVDISSNGED WISLKEGNKA IIFQGNTNPT DVVLGVFSKP
NP_659566_1_neu TKKKYYVKTY RVDISSNGED WITLKEGNKA IIFQGNTNPT DVVFGVFPKP
NP_990113_1_neu TKKEYYLKTY RVDVSSNGED WITLKEGNKP VVFQGNSNPT DVVYRPFPKP
NP_001093692_1_ TKKKYFVKSY KVDISSNGED WITLKDGNKH LVFAGNTDAN DVVYRAFPKP
NP_852474_2_neu TKKKYYVKEY KVDVSSNGED WITIKDGPKQ KLFQGNTNPT DVVKAKFPKP
Consistency     *9*9*99*7* 89*9****** **98*8*8*5 78*7**9988 ***456*8**

                . . . . . . . . . 410. . . . . . . . . 420. . . . . . . . . 430. . . . . . . . . 440. . . . . . . . . 450
NP_003864_5_neu LITRFVRIKP ATWETGISMR FEVYGCKITD YPCSGMLGMV SGLISDSQIT
XP_001143690_1_ LITRFVRIKP ATWETGISMR FEVYGCKITD YPCSGMLGMV SGLISDSQIT
NP_001252745_1_ LITRFVRIKP ATWETGISLR FEVYGCKITD YPCSGMLGMV SGLISDSQIT
NP_001192589_1_ LITRFVRIKP VTWETGISMR FEVYGCKITD YPCSGMLGMV SGLISDSQIT
XP_005617003_1_ LITRFVRIKP MTWETGISMR FEVYGCKITD YPCSGMLGMV SGLISDSQIT
NP_032763_2_neu LITRFVRIKP VSWETGISMR FEVYGCKITD YPCSGMLGMV SGLISDSQIT
NP_659566_1_neu LITRFVRIKP ASWETGISMR FEVYGCKITD YPCSGMLGMV SGLISDSQIT
NP_990113_1_neu VLTRFVRIKP VSWENGVSLR FEVYGCKITD YPCSGMLGMV SGLIPDSQIT
NP_001093692_1_ VITRFVRIRP VTWENGISLR FELYGCKITD YPCSRMLGMV SGLISDSQIT
NP_852474_2_neu TLTRYPRIRP INWETGIALR FEVYGCKISE YPCSGMLGMV SGLITDSQIT
Consistency     79**98**8* 67**7*998* **9*****99 ****8***** ****7*****

                . . . . . . . . . 460. . . . . . . . . 470. . . . . . . . . 480. . . . . . . . . 490. . . . . . . . . 500
NP_003864_5_neu SSNQGDRNWM PENIRLVTSR SGWALPPA-P HSYINEWLQI DLGEEKIVRG
XP_001143690_1_ SSNQGDRNWM PENIRLVTSR SGWALPPA-P HSYVNEWLQI DLGEEKIVRG
NP_001252745_1_ SSNQGDRNWM PENIRLVTSR SGWALPPA-P HSYVNEWLQI DLGEEKIVRG
NP_001192589_1_ ASNQADRNWM PENIRLVTSR SGWALPPA-P HPYVNEWLQV DLAEEKIVRG
XP_005617003_1_ ASNQGERNWM PENIRLVTSR SGWTLPPA-P HPYINEWLQV DLGEEKIVRG
NP_032763_2_neu ASNQADRNWM PENIRLVTSR TGWALPPS-P HPYTNEWLQV DLGDEKIVRG
NP_659566_1_neu ASNQGDRNWM PENIRLVTSR TGWALPPS-P HPYINEWLQV DLGDEKIVRG
NP_990113_1_neu ASTQVDRNWI PENARLITSR SGWALPPT-T HPYTNEWLQI DLGEEKIVRG
NP_001093692_1_ ASSQVDRNWV PELARLVTSR SGWALPPSNT HPYTKEWLQI DLAEEKIVRG
NP_852474_2_neu VSSHIERTWV SENARLMTSR SGWMLLPQ-S QPYADEWLQI DLAEEKLVKG
Consistency     7*7848*8*8 8*87**8*** 8**7*8*606 86*67****9 **78**9*9*

                . . . . . . . . . 510. . . . . . . . . 520. . . . . . . . . 530. . . . . . . . . 540. . . . . . . . . 550
NP_003864_5_neu IIIQGGKHRE NKVFMRKFKI GYSNNGSDWK MIMDDSKRKA KSFEGNNNYD
XP_001143690_1_ IIIQGGKHRE NKVFMRKFKI GYSNNGSDWK MIMDDSKRKA KSFEGNNNYD
NP_001252745_1_ IIIQGGKHRE NKVFMRKFKI GYSNNGSDWK MIMDDSKRKA KSFEGNNNYD
NP_001192589_1_ VIIQGGKHRE NKVFMRKFKI GYSNNGSDWK MIMDDSKRKA KSFEGNNNYD
XP_005617003_1_ IIIQGGKHRE NKVFMRKFKI GYSNNGSDWR MIMDDSKRKA KSFEGNNNYD
NP_032763_2_neu VIIQGGKHRE NKVFMRKFKI AYSNNGSDWK TIMDDSKRKA KSFEGNNNYD
NP_659566_1_neu VIIQGGKHRE NKVFMRKFKI AYSNNGSDWK MIMDDSKRKA KSFEGNNNYD
NP_990113_1_neu IIVQGGKHRE NKVFMKKFKI GYSNNGSDWK MIMDSSKKKI KTFEGNTNYD
NP_001093692_1_ VIIQGGKHKE NKVFMRKFKI GYSNNGTEWE MIMDSSRNKP KTFEGNTNYD
```

```
NP_852474_2_neu LIIQGGKHRD NKVFMKKFRL GYSNNGSDWK LAMDATGNKP KIFEGNLNYD
Consistency     8*9*****99 *****8**99 8*****99*8 88**6977*6 *7****6***

                .........560.........570.........580.........590.........600
NP_003864_5_neu TPELRTFPAL STRFIRIYPE RATHGGLGLR MELLGCEVEA PTAGPTTPNG
XP_001143690_1_ TPELRTFPAL STRFIRIYPE RATHGGLGLR MELLGCEVEA PTAGPTTPNG
NP_001252745_1_ TPELRTFPAL STRFIRIYPE RATHGGLGLR MELLGCEVEA PTAGPTTPNG
NP_001192589_1_ TPELRTFPPL STRFIRIYPE RATHGGLGLR MELLGCEVEA PTAGPTTPNG
XP_005617003_1_ TPELRTFPPL STRFIRIYPE RATHGGLGLR MELLGCEVEA PTAGPTTPNG
NP_032763_2_neu TPELRTFSPL STRFIRIYPE RATHSGLGLR MELLGCEVEA PTAGPTTPNG
NP_659566_1_neu TPELRAFTPL STRFIRIYPE RATHSGLGLR MELLGCEVEV PTAGPTTPNG
NP_990113_1_neu TPELRTFEPV STRIIRVYPE RATHAGLGLR MELLGCELEA PTAVPTVSEG
NP_001093692_1_ TPELRTFAPI TTRFIRIIPE RASVSGLGLR LELLGCEVET PTSIPTTPEV
NP_852474_2_neu TPALRTMEPV LTRFVRIYPD RGTPAGMGLR LELLGCEMEV PTVPPTTPAA
Consistency     **8**88568 7**89*98*9 *8966*9*** 8******8*7 **85**8867

                .........610.........620.........630.........640.........650
NP_003864_5_neu N--LVDECDD DQANCHSGTG DDFQLTGGTT VLATEKPTVI DSTIQSEFPT
XP_001143690_1_ N--LVDECDD DQANCHSGTG DDFQLTGGTT VLATEKPTVI DSTIQSEFPT
NP_001252745_1_ N--PVDECDD DQANCHSGTG DDFQLTGGTT VLATEKPTVI DSTIQSEFPT
NP_001192589_1_ N--LVDECDD DQANCHSGTG DDFQLTGGTT VLTTEKPTVI DSTIQSEFPT
XP_005617003_1_ N--LVDECDD DQANCHSGTG DDFQLTGGTT VLTTERPTVI DSTIQSEFPT
NP_032763_2_neu N--PVDECDD DQANCHSGTG DDFQLTGGTT VLATEKPTII DSTIQSEFPT
NP_659566_1_neu N--PVDECDD DQANCHSGTG DDFQLTGGTT VLATEKPTII DSTIQSEFPT
NP_990113_1_neu K--PVDECDD DQANCHSG-- -----TGGTT VLNTEKPTVI DNTVQPELPP
NP_001093692_1_ N--GMDECED DLANCHSGTD EGFKLTVGTT AQSTETPTVE ASPEEPDMTH
NP_852474_2_neu STTPSDECDD DQANCHSGTG DGYDQTGGTT --ATE--TIR E---MSTIPA
Consistency     80047***9* *8******86 75766*8*** 766**68*96 7766878786

                .........660.........670.........680.........690.........700
NP_003864_5_neu Y-GFNCEFGW GSHKTFCHWE HDNHVQLKWS VLTS------K TGPIQDHT-G
XP_001143690_1_ Y-GFNCEFGW GSHKTFCHWE HDNHVQLKWS VLTS------K TGPIQDHT-G
NP_001252745_1_ Y-GFNCEFGW GSHKTFCHWE HDNHVQLKWS VLTS------K TGPIQDHT-G
NP_001192589_1_ Y-GFNCEFGW GSHKTFCHWE HDNHVQLKWS VLTS------K TGPIQDHT-G
XP_005617003_1_ Y-GFNCEFGW GSHKTFCRWE HDNQVQLKWS VLTS------K TGPIQDHTAG
NP_032763_2_neu Y-GFNCEFGW GSHKTFCHWE HDSHAQLRWS VLTS------K TGPIQDHT-G
NP_659566_1_neu Y-GFNCEFGW GSHKTFCHWE HDSHAQLRWR VLTS------K TGPIQDHT-G
NP_990113_1_neu Y-NLNCGFGW GSHKTLCQWE HDNQVDLKWA ILTS------K TGPIQDHT-G
NP_001093692_1_ T-DLDCKFGW GSHKTLCNWE HDIGSDLKWA VLNS------K TGPVQDHT-G
NP_852474_2_neu FLWFACDFGW ANDPSFCGWI SED-SGFRWQ IQSSGTPTLN TGPNMDHTGG
Consistency     70687*7*** 898898*5*8 8964668*6 988*000008 ***88***0*

                .........710.........720.........730.........740.........750
NP_003864_5_neu DGNFIYSQAD ENQKGKVARL VSPVVYSQNS AHCMTFWYHM SGSHVGTLRV
XP_001143690_1_ DGNFIYSQAD ENQKGKVARL VSPVVYSQNS AHCMTFWYHM SGSHVGTLRV
NP_001252745_1_ DGNFIYSQAD ENQKGKVARL VSPVVYSQNS AHCMTFWYHM SGSHVGTLRV
NP_001192589_1_ DGNFIYSQAD ENQKGKVARL VSPVVYSQNA AHCMTFWYHM SGSHVGTLRV
XP_005617003_1_ DGNFIYSQAD ENQKGKVARL VSPVVYSQNS AHCMTFWYHM SGSHVGTLRV
NP_032763_2_neu DGNFIYSQAD ENQKGKVARL VSPVVYSQSS AHCMTFWYHM SGSHVGTLRV
NP_659566_1_neu DGNFIYSQAD ENQKGKVARL VSPVVYSQSS AHCMTFWYHM SGSHVGTLRV
NP_990113_1_neu DGNFIYSQAD ESQKGKVARL LSPIIYSQNS AHCMTFWYHM SGPHVGTLKI
NP_001093692_1_ DGNFIYSEVD ERHEGRTARL MSPVVSSARS AHCMTFWYHM DGSHVGTLSI
NP_852474_2_neu SGNFIYTLAT GAQETEVARL VSPSVSGQDS DLCLSFWYHM FGSHIGTLHI
Consistency     8*****9798 868888***  8**897886 9 88*99***** 7*8*9***79

                .........760.........770.........780.........790.........800
NP_003864_5_neu KLRYQKPE-E YDQLVWMAIG HQGDHWKEGR VLLHKSLKLY QVIFEGEIGK
XP_001143690_1_ KLRYQKPE-E YDQLVWMAIG HQGDHWKEGR VLLHKSLKLY QVIFEGEIGK
NP_001252745_1_ KLRYQKPE-E YDQLVWMAIG HQGDHWKEGR VLLHKSLKLY QVIFEGEIGK
NP_001192589_1_ KLRYQKPE-E YDQLVWMAIG HQGDHWKEGR VLLHKSLKLY QVIFEGEIGK
XP_005617003_1_ KLHYQKPE-E YDQLVWMAIG HQGDHWKEGR VLLHKSLKLY QVIFEGEIGK
NP_032763_2_neu KLRYQKPE-E YDQLVWMVVG HQGDHWKEGR VLLHKSLKLY QVIFEGEIGK
NP_659566_1_neu KLHYQKPE-E YDQLVWMVVG HQGDHWKEGR VLLHKSLKLY QVIFEGEIGK
NP_990113_1_neu KLRYQKPD-E YDQVLLSLNG HQANCWQEGR VLLHKSVKLY QVVIEGEIGK
NP_001093692_1_ KLKYEMEE-G FDQTLWTLSG NQGDQWKEAR VLLHKTMKQY RVIVEGTVGK
NP_852474_2_neu KQRRETSQGS ADVLLWTVSG HQGNRWREGR VLIPHSNKPY QVIIESVVER
Consistency     *87887780 7 7*888665* 8*886*8*8* **98897*6* 9*97*87989

                .........810.........820.........830.........840.........850
NP_003864_5_neu GNLGGIAVDD ISINNHISQE DCAKPA--DL DKK--NPEIK IDE-TGSTPG
XP_001143690_1_ GNLGGIAVDD ISINNHISQE DCAKPA--DL DKK--NPEIK IDE-TGSTPG
NP_001252745_1_ GNLGGIAVDD ISINNHISQE DCAKPA--DL DKK--NPEIK IDE-TGSTPG
NP_001192589_1_ GNLGGIAVDD ISINNHIPQE DCAKPA--DL DKK--NPESK IDE-TGSTPG
XP_005617003_1_ GNLGGIAVDD ISINNHISQE DCAKPS--DL DKR--NPENK IDE-TGSTPG
NP_032763_2_neu GNLGGIAVDD ISINNHISQE DCAKPT--DL DKK--NTEIK IDE-TGSTPG
```

```
NP_659566_1_neu GNLGGIAVDD ISINNHIPQE DCAKPT--DL DKK--NTEIK IDE-TGSTPG
NP_990113_1_neu GN-GGIAVDD INIDNHISQE DCQKST--DV ESEIVEEDPE SNQ-TGFTPS
NP_001093692_1_ GSTGGIAVDD IIIANHISTS HCRTPEIDDS SNKIIEENSE IDK-TGSTPN
NP_852474_2_neu KSWGDIAVDD IKILDNVNMA DCKDP---DV PAEPIQPEDN INEIMVDITD
Consistency     885*8***** *6*6989677 8*678400*7 7670075847 8880887886

                .........860.......870.......880.......890.......900
NP_003864_5_neu YEGEGEGDKN ISRKPGNVLK TLDPILITII AMSALGVLLG AVCGVVLYCA
XP_001143690_1_ YEGEGEGDKN ISRKPGNVLK TLDPILITII AMSALGVLLG AVCGVVLYCA
NP_001252745_1_ YEGEGEGDKN ISRKPGNVLK TLDPILITII AMSALGVLLG AVCGVVLYCA
NP_001192589_1_ YQGAGEGDEN ISRKPGNVLK TLDPILITII AMSALGVLLG AVCGVVLYCA
XP_005617003_1_ YEGTGEGDEN ISRKPGNVLK TLDPILITII AMSALGVLLG AVCGVVLYCA
NP_032763_2_neu YEGEGEGDKN ISRKPGNVLK TLDPILITII AMSALGVLLG AVCGVVLYCA
NP_659566_1_neu YE-EGKGDKN ISRKPGNVLK TLDPILITII AMSALGVLLG AVCGVVLYCA
NP_990113_1_neu YRTDEDYD-D ISRKPGNVLK TLDPILITII AMSALGVLLG AICGVVLYCA
NP_001093692_1_ Y-ALNEFNES ISKKPGNVLK TLDPILITII AMSALGVLLG AICGVVLYCA
NP_852474_2_neu FPDIVE-NPD IG-GAGNMLK TLDPILITII AMSALGVFLG AICGVVLYCA
Consistency     9544585857 *8788**9** ********** *******8** *9********

                .........910.......920.......930.......940.
NP_003864_5_neu CWHNGMSERN LSALENYNFE LVDGVKLKKD KLNTQSTYSE A
XP_001143690_1_ CWHNGMSERN LSALENYNFE LVDGVKLKKD KLNTQSTYSE A
NP_001252745_1_ CWHNGMSERN LSALENYNFE LVDGVKLKKD KLNTQSTYSE A
NP_001192589_1_ CWHNGMSERN LSALENYNFE LVDGVKLKKD KLNPQSTYSE A
XP_005617003_1_ CWHNGMSERN LSALENYNFE LVDGVKLKKD KLNTQSTYSE A
NP_032763_2_neu CWHNGMSERN LSALENYNFE LVDGVKLKKD KLNPQSNYSE A
NP_659566_1_neu CWHNGMSERN LSALENYNFE LVDGVKLKKD KLNPQSNYSE A
NP_990113_1_neu CWHNGMSERN LSALENYNFE LVDGVKLKKD KLNTQNSYSE A
NP_001093692_1_ CWHNGMSERN LSALENYNFE LVDGVKLKKD KLNTQNSYSE A
NP_852474_2_neu CSHSGMSDRN LSALENYNFE LVDGVKLKKD KLNSQNSYSE A
Consistency     *8*9***9** ********** ********** ***6*76*** *
```
